# Comparative case study of evolutionary insights and floral complexity in key early-diverging eudicot Ranunculales models

**DOI:** 10.3389/fpls.2024.1486301

**Published:** 2024-10-30

**Authors:** Bharti Sharma, Mankirat Kaur Pandher, Ana Quetzali Alcaraz Echeveste, Marianellie Bravo, Rene Kenny Romo, Sarah Christine Ramirez

**Affiliations:** Department of Biological Sciences, California State Polytechnic University, Pomona, CA, United States

**Keywords:** Ranunculales, floral evolution, evo-devo, petal elaboration, organ identity, key model systems

## Abstract

Famously referred to as “Darwin’s abominable mystery,” the rapid diversification of angiosperms over the last ~140 million years presents a fascinating enigma. This diversification is underpinned by complex genetic pathways that evolve and rewire to produce diverse and sometimes novel floral forms. Morphological innovations in flowers are shaped not only by genetics but also by evolutionary constraints and ecological dynamics. The importance of model organisms in addressing the long-standing scientific questions related to diverse floral forms cannot be overstated. In plant biology, *Arabidopsis thaliana*, a core eudicot, has emerged as a premier model system, with its genome being the first plant genome to be fully sequenced. Similarly, model systems derived from crop plants such as *Oryza sativa* (rice) and *Zea mays* (maize) have been invaluable, particularly for crop improvement. However, despite their substantial utility, these model systems have limitations, especially when it comes to exploring the evolution of diverse and novel floral forms. The order Ranunculales is the earliest-diverging lineage of eudicots, situated phylogenetically between core eudicots and monocots. This group is characterized by its exceptional floral diversity, showcasing a wide range of floral morphologies and adaptations that offer valuable insights into the evolutionary processes of flowering plants. Over the past two decades, the development of at least five model systems including, *Aquilegia*, *Thalictrum*, *Nigella*, *Delphinium* and *Eschscholzia* within the Ranunculales order has significantly advanced our understanding of floral evolution. This review highlights the conservation and divergence of floral organ identity programs observed among these models and discusses their importance in advancing research within the field. The review also delves into elaborate petal morphology observed in *Aquilegia*, *Nigella*, and *Delphinium* genera, and further discusses the contributions, limitations, and future research directions for Ranunculales model systems. Integrating these diverse models from the early-diverging eudicot order has enhanced our understanding of the complex evolutionary pathways that shape floral diversity in angiosperms, bridging the knowledge gaps essential for a comprehensive understanding of floral evolution.

## 1 Introduction

The evolution of flower morphology is a multifaceted and nuanced process shaped by the interplay of genetic, environmental, ecological, and evolutionary factors (Becker et al., 2011). This complex interaction has given rise to the remarkable diversity of floral forms observed in angiosperms, which have diversified into over 350,000 species (Endress, 2011; Moyroud and Glover, 2017). These diverse species of angiosperms are categorized into major clades, including early-diverging angiosperms (ANA clade), magnoliids, monocots, early-diverging eudicots, and core eudicots, based on a combination of morphological, genetic, phylogenetic, and evolutionary criteria (Soltis and Soltis, 2013; Li et al., 2021).

Over the past three decades, research on floral organ identity has been fundamental in elucidating the mechanisms driving the evolution of floral morphology. Central to this body of research is the seminal ABC model, established more than thirty years ago (Coen and Meyerowitz, 1991). This model was initially developed through studies on two core eudicot model systems, *Arabidopsis thaliana*, a member of the Brassicaceae family from the Rosid clade, and *Antirrhinum majus*, belonging to the Plantaginaceae family from the Asterid clade. The ABC model was later expanded to the more comprehensive ABCDE model (Colombo et al., 1995; Pelaz et al., 2000), which provides a framework for understanding how floral organ identities are specified through the combinatorial function of A, B, C, D, and E class genes. According to this model, the A+E specifies sepal identity, A+B+E specifies petal identity, B+C+E specifies stamen identity, C+E specifies carpel identity, and the D class genes are crucial for ovule identity (Bowman et al., 1991; Ma and dePamphilis, 2000; Soltis et al., 2006).

**Figure 1:**
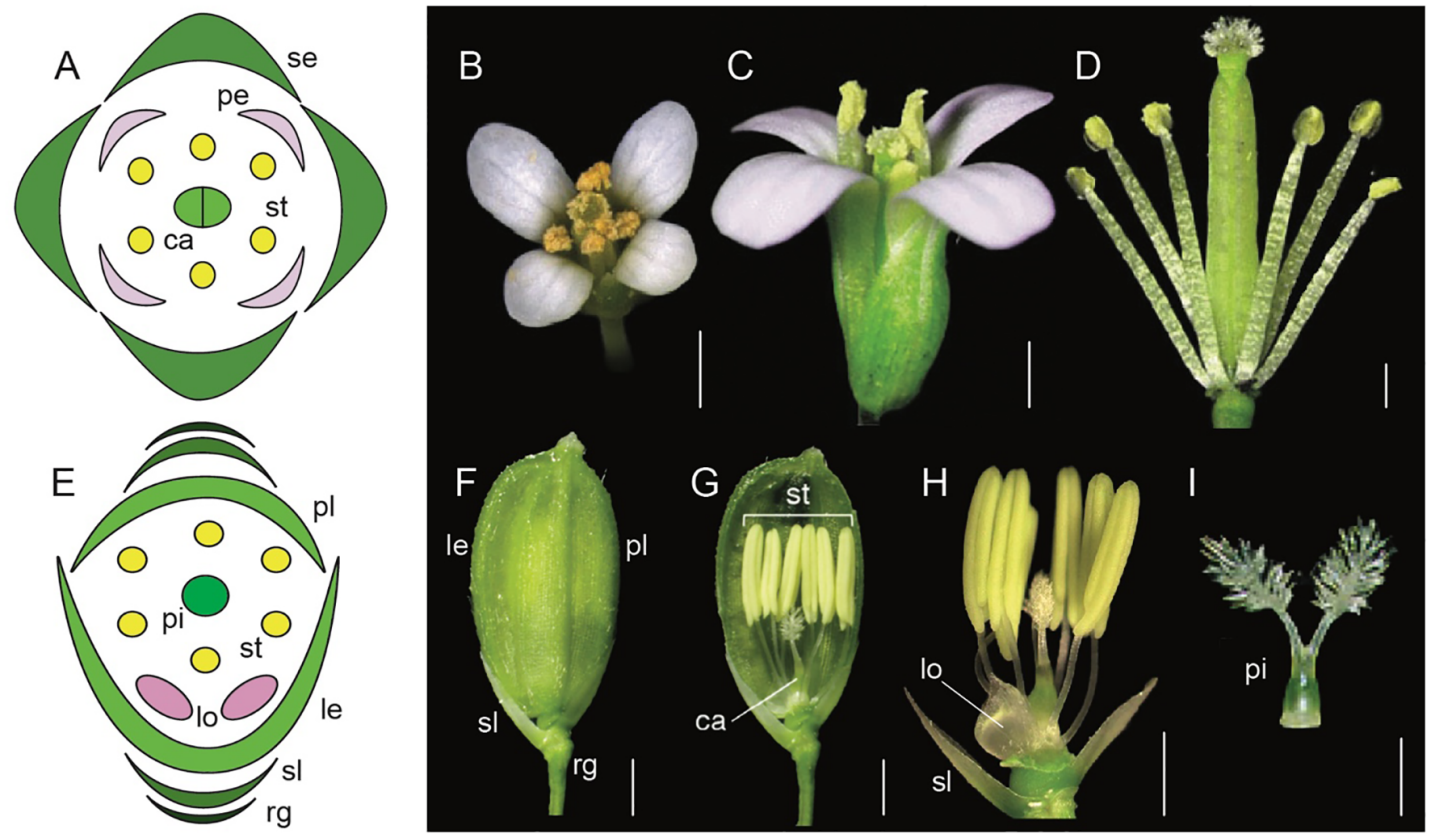
Comparative floral morphology of *Arabidopsis thaliana* and *Oryza sativa* (Adapted from Xue et al., 2020; Krämer, 2015; Tanaka et al., 2017; Sugiyama et al., 2019; Dreni, 2023; Logacheva et al., 2008; Hirano et al., 2014). **(A)** *A. thaliana* floral diagram **(B)** *A. thaliana* wild-type flower top view **(C)** *A. thaliana* wild-type side view **(D)** *A. thaliana* androecium and gynoecium attached to the receptacle **(E)** *Oryza sativa* floral diagram **(F)** mature *O. sativa* spikelet (rg, rudimentary glume; sl, sterile lemma; le, lemma; pl, palea) **(G)** *O. sativa* androecium, gynoecium, sterile lemma and lodicules attached to the receptacle **(H)** *O. sativa* syncarpic ovary, stamens, lodicule, and sterile lemma attached to the receptacle. **(I)** *O. sativa* syncarpic ovary. Scale bars: **(B)** 1 mm, **(C)** 200 μm, **(D)** 500 μm, **(F)** 1 mm, **(G)** 1 mm, **(H)** 1 mm, **(I)** 1 mm.

The flower structure of *A. thaliana* exemplifies a typical simple flower, consisting of four concentric whorls: sepals (4), petals (4), stamens (6), and carpels (2-fused) (**Figures 1A–D**). Over the years, *A. thaliana* has become an impressive model system, it has a short life cycle, and availability of many functional tools including stable transformations, and a small genome size (Meyerowitz and Pruitt, 1985). The genome of *Arabidopsis thaliana* was sequenced nearly a quarter century ago in 2000, marking it the first plant genome to be fully sequenced (The Arabidopsis Genome Initiative, 2000) The simplicity of this floral structure and ease of genetic work has made *Arabidopsis* an indispensable core eudicot model system.

Equally remarkable has been the research in monocots with model systems like *Oryza sativa* (rice), *Zea mays* (maize), and *Sorghum bicolor* (sorghum). Flowers in grasses have perianth organs that are morphologically distinct from those in core eudicots. For example, rice flowers contain sterile organs the lemma (1), palea (1), and lodicules (2), as well as fertile organs stamens (6), and a syncarpic ovary with two styles and a feathery stigma (**Figures 1E–I**, Yoshida and Nagato, 2011; Dreni, 2023). Many aspects of the ABC model are conserved, however there are also unique genetic players in monocots that fulfill the A function differently compared to *Arabidopsis.* This highlights the evolutionary diversification of floral organ identity mechanisms across angiosperms.

**Figure 2:**
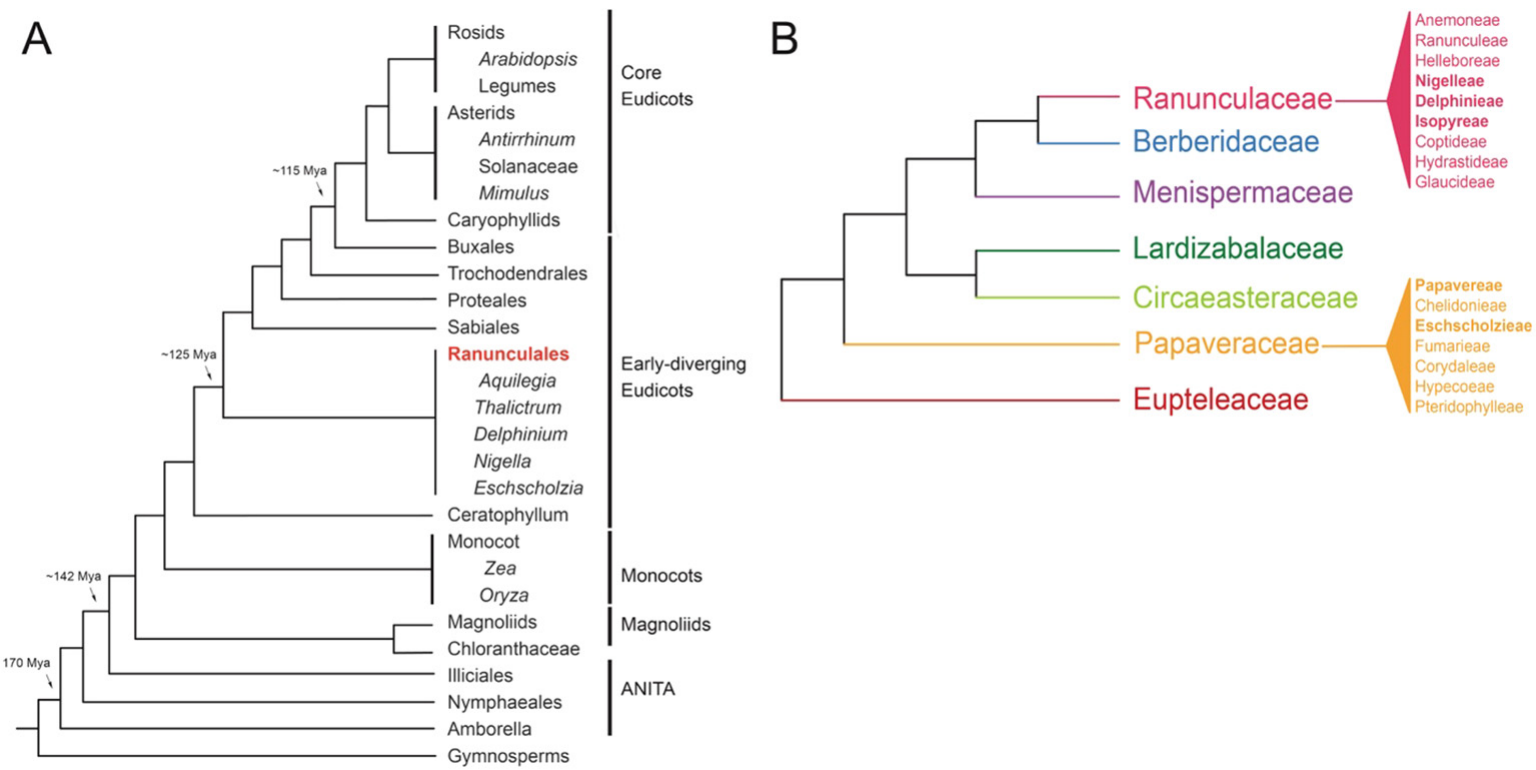
Phylogenies **(A)** Simplified angiosperm phylogeny based on Moore et al., 2007 and adapted from Kramer, 2009, showing genera with notable resources available **(B)** Simplified Ranunculales phylogeny, showing tribes within the Raunculaceae and Papaveraceae, based on The RanOmics group et al., 2024; Delpeuch et al., 2022; Zhai et al., 2019; Peng et al., 2023; Peng et al., 2024.

While *Arabidopsis* and rice have been indispensable model organisms for research, addressing comprehensive evolutionary inquiries, particularly pertaining to the origins and diversification of distinct floral structures and observed developmental pathways, is limited. The incorporation of early-diverging eudicots, such as those within the Ranunculales order, into eco-evo-devo studies has offered critical insights into floral organ evolution. Positioned at the evolutionary midpoint between core eudicots and monocots, several genera within Ranunculales, such as *Aquilegia*, *Thalictrum, Nigella*, *Delphinium*, and *Eschscholzia*, serve as excellent model systems for studying floral development and organ identity (**Figures 2A, B**, The RanOmics group et al., 2024). These genera exhibit a range of floral morphologies that provide insights into the evolutionary and genetic mechanisms underlying flower development.

One of the central themes of evo-devo studies is understanding morphological evolution, particularly within the scope of organ identity establishment. What are the genetic factors and developmental pathways that drive the formation and diversification of floral organs? Studies in these early-diverging eudicots within the framework of the ABC model not only expand the taxonomic breadth of research but also provide comparative data crucial for deciphering evolutionary transitions in floral morphology. By bridging insights from core eudicots and monocots with those from early-diverging eudicots, genomic researchers can uncover conserved genetic networks and adaptive innovations that have shaped the remarkable diversity of angiosperm flowers. This review comprehensively focuses on morphological innovations and organ identity research in the major emerging model systems in Ranunculales, with a particular focus on the petal elaborations in genera *Aquilegia*, *Nigella*, and *Delphinium*. We also discuss how gene duplications have influenced the evolution of floral forms within the framework of the ABC model. Additionally, the review seeks to highlight the significance of these model systems in understanding broader evolutionary patterns and mechanisms in angiosperms.

## 2 Morphological diversity and distribution

**Figure 3:**
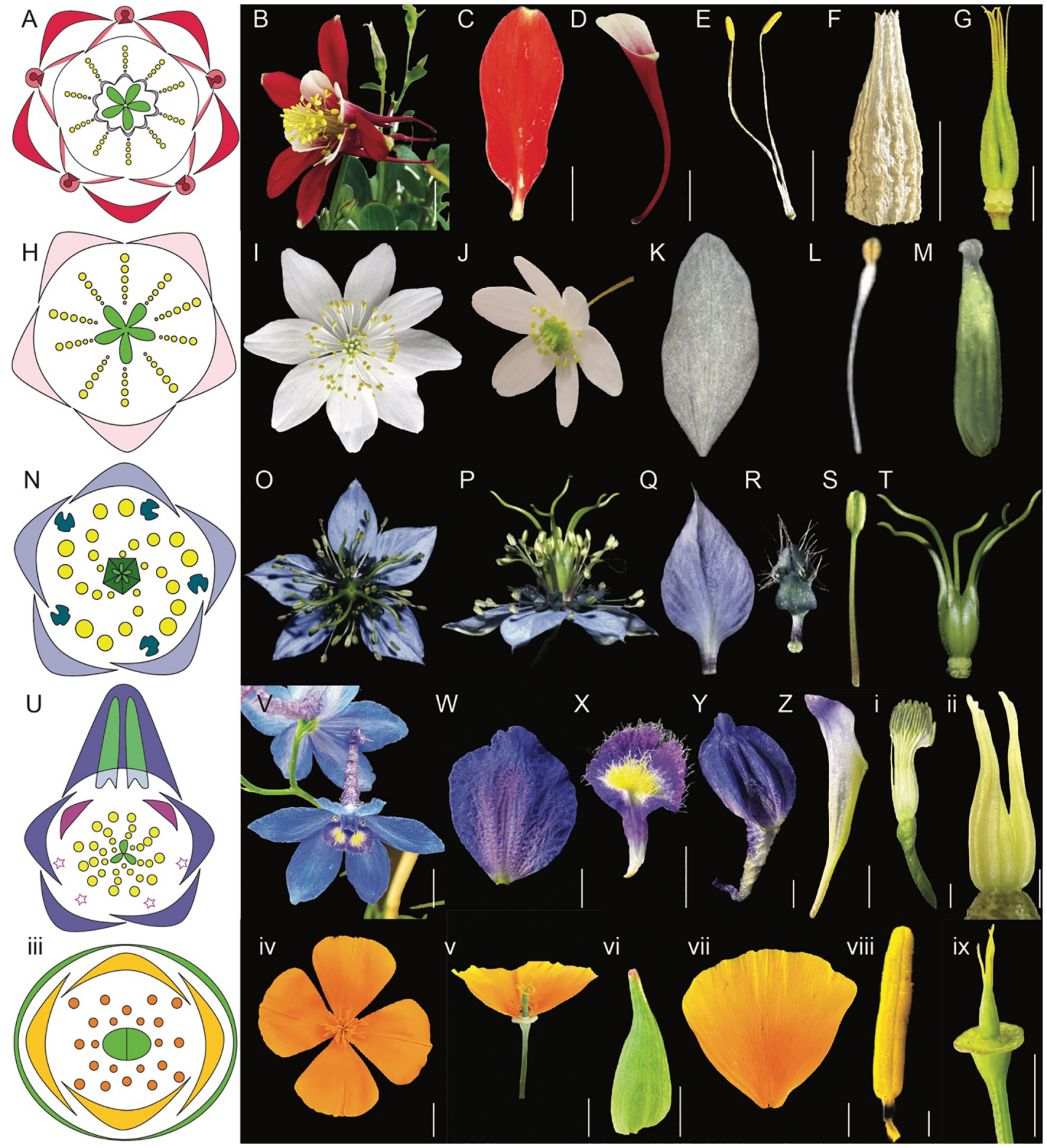
Comparative morphology of five Ranunculales models, *Aquilegia*, *Thalictrum*, *Nigella*, *Delphinium*, and *Eschscholzia* (Adapted from Sharma et al., 2024; Sharma et al., 2014; Galimba et al., 2012; Arias et al., 2021; Wang et al., 2015; Jabbour et al., 2009; Becker et al., 2023; Di Stilio et al., 2009). **(A–G)** *Aquilegia coerulea* **(A)** floral diagram **(B)** flower side view **(C)** sepal **(D)** spurred petal **(E)** mature stamens **(F)** staminodia **(G)** carpels attached to receptacles **(H–M)** *Thalictrum* **(H)** floral diagram **(I)** flower top view **(J)** flower side view **(K)** sepal **(L)** stamen **(M)** carpel **(N–T)** *Nigella damascena* **(N)** floral diagram **(O)** flower top view **(P)** flower side view **(Q)** sepal **(R)** petal **(S)** stamen **(T)** carpels attached to receptacle **(U-ii)** *Delphinium x belladona* ‘Bellamosum’ **(U)** floral diagram **(V)** flower top view **(W)** sepal **(X)** lateral petal **(Y)** dorsal spurred sepal **(Z)** spurred petal i. stamens attached to receptacle ii. carpels attached to receptacle iii-ix *Eschscholzia californica* iii. floral diagram iv. flower top view v. flower side view vi. sepal vii. petal viii. anther ix. carpel attached to receptacle. Scale bars: **(B)** 1 cm, **(C)** 5 mm, **(D)** 1 cm, **(E)** 5 mm, F. 5 mm, **(G)** 5 mm, **(W)** 5 mm, **(X)** 5 mm, **(Y)** 5 mm, **(Z)** 5 mm, ii. 1 mm, iv. 1 cm, v. 1 cm, vi. 5 mm, vii. 5 mm, viii. 1 mm, ix. 5 mm (The images for *Thalictrum* **(H–M)** and *Nigella* **(N–T)** are adapted from the sources mentioned above. Since the original pictures did not have scale bars, we were unable to provide that information in this figure).

The species-rich order Ranunculales, which emerged ~115 million years ago (Mya), consists of approximately 4500 species distributed in seven families, including Ranunculaceae (~2500 sp), Berberidaceae (~700 sp), Menispermaceae (~440 sp), Lardizabalaceae (~40 sp), Circeasteraceae (~2 sp), Papaveraceae (~430-825), and Eupteleaceae (~2 sp) (**Figure 2B**, Damerval and Becker, 2017; The RanOmics group et al., 2024). Several genera within the Ranunculales order have emerged as valuable model systems for studying floral development and organ identity. Notably, *Aquilegia*, *Thalictrum*, *Nigella*, *Delphinium*, and *Eschscholzia* from the Ranunculales order, offer diverse floral morphologies that provide crucial insights into the genetic mechanisms driving flower development (Kramer et al., 2007; Drea et al., 2007; Di Stilio et al., 2010; Jabbour and Renner, 2012b; Gonçalves et al., 2013; Wang et al., 2015; Zhao et al., 2023). In the following section, we discuss the geographical distribution and distinctive floral morphology of each model system offering a concise overview of their evolution and adaptation to diverse environments and pollinators (**Figures 3A-ix**, **Supplementary Table 1**).

### 2.1 Aquilegia

The genus *Aquilegia*, consisting of around 70-80 species (Munz, 1946; Whittemore and Parfitt, 1997; Nold, 2003), is a classic example of adaptive radiation (Hodges and Derieg, 2009; Kramer, 2009; Kramer and Hodges, 2010; Sharma et al., 2014). *Aquilegia* originated in Eastern Asia around 6 to 6.9 Mya and subsequently diversified into Europe and North America between 1 to 3 Mya. This diversification involved two distinct radiation events, leading to the development of species in Europe and North America (Bastida et al., 2010; Fior et al., 2013). The genus is now distributed across Asia (23 species), Europe (21 species), and North America (22 species) (Bastida et al., 2010). Interestingly, the diversification of *Aquilegia* was primarily driven by allopatric speciation through geographic isolation in Europe (Bastida et al., 2010). In contrast, sympatric speciation, likely influenced by pollinator specialization, played a significant role in the diversification of species in North America. This divergence in speciation mechanisms underscores the contrasting evolutionary pressures experienced by the genus in these two regions (Bastida et al., 2010). These distinct evolutionary trajectories position *Aquilegia* as a key model genus for exploring adaptive radiation and speciation processes.

The actinomorphic perianth of *Aquilegia* comprises five types of floral organs (**Figures 3A, B**). The outermost whorl consists of petaloid sepals (5) (**Figure 3C**). The second whorl includes nectariferous, spurred petals (5) (**Figure 3D**, Ren et al., 2011). Inner to the petals are stamens, in 10 orthostichies, that are either opposite to sepals or petals (**Figure 3E**, Tucker and Hodges, 2005; Sharma et al., 2014). The fourth organ identity features the novel organ, thin and papery staminodes arranged in two whorls of five surrounding the gynoecium, consisting of 10 sterile structures (**Figure 3F**). The innermost whorl comprises unfused green carpels (4-7) (**Figure 3G**, Sharma et al., 2014), with the ovary being broader and tapering into a straight style.

**Figure 4:**
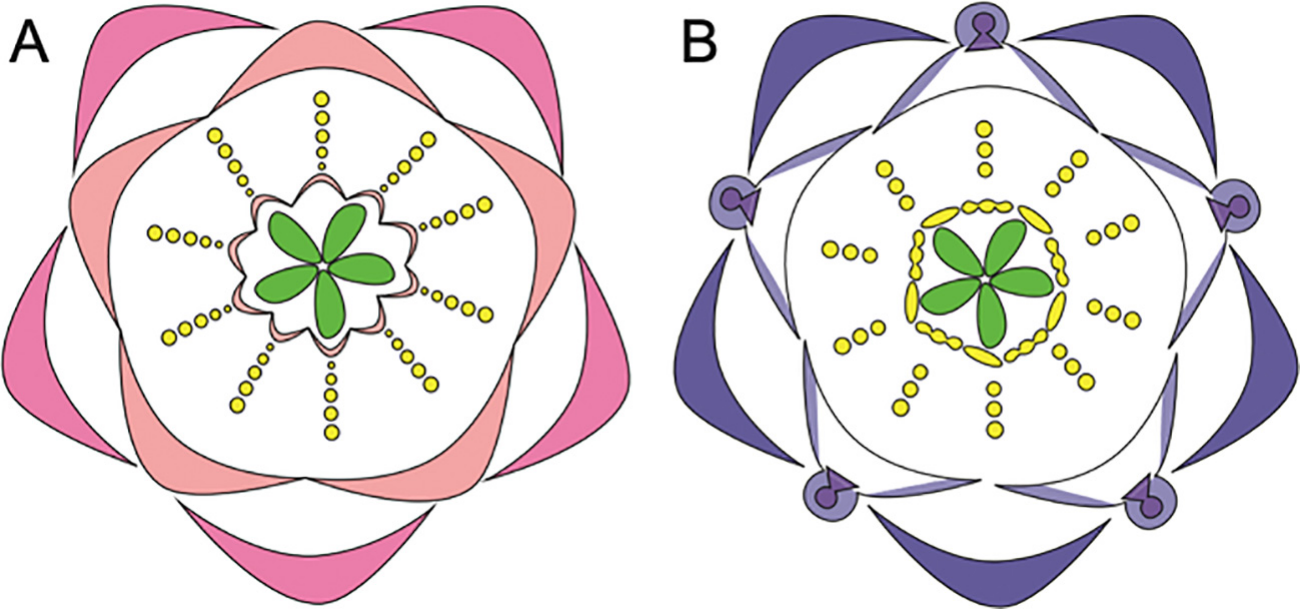
Floral diagrams illustrating species with variations from the typical *Aquilegia* perianth structure **(A)** *A. ecalcarata*, lacks spurs and **(B)** *A. jonesii*, lacks staminodia (Adapted from Tucker and Hodges, 2005; Kramer, 2009; Johns et al., 2024).

**Figure 5:**
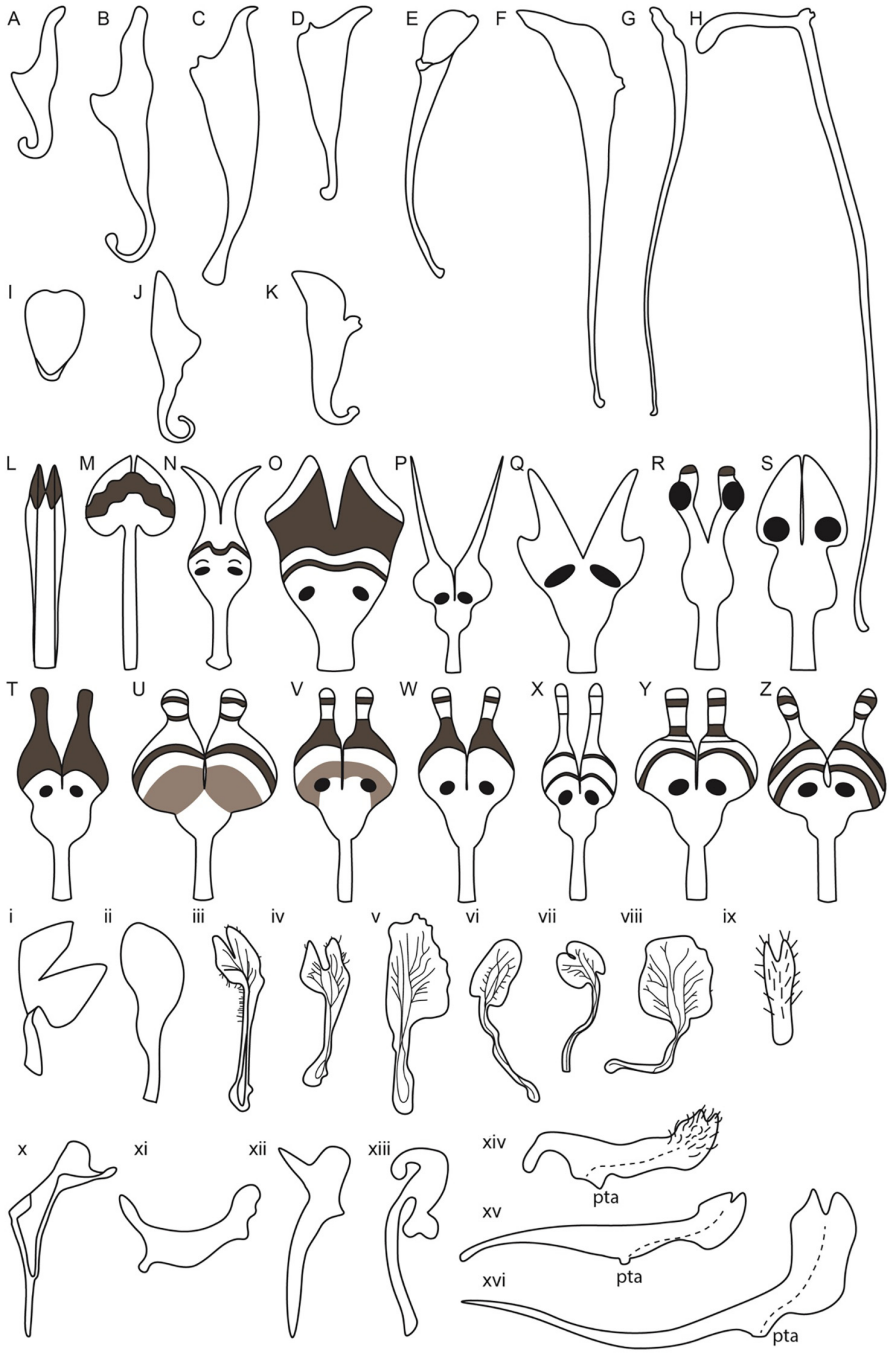
Petal diversity of Ranunculales models, *Aquilegia*, *Nigella*, *Delphinium* along with related Delphinieae species, *Staphisagria* and *Aconitum*. (Adapted from Ballerini et al., 2019; Edwards et al., 2022; Munz, 1967; Kramer and Hodges, 2010; Bosch et al., 1997; Zhang et al., 2022; Delpeuch et al., 2022; Yao et al., 2019) *Aquilegia* petals **(A–K) (A)** *A. brevistyla* **(B)** *A. saximontana* **(C)** *A. canadensis* **(D)** *A. formosa* **(E)** *A. coerulea* **(F)** *A. chrysantha* **(G)** *A. pinetorum* **(H)** *A. longissima* **(I)** *A. ecalcarata* **(J)** *A. flabellata* **(K)** *A. sibirica*; *Nigella* petals from simple to complex **(L–Z) (L)** *N. integrifolia* **(M)** *N. unguicularis* **(N)** *N. nigellastrum* **(O)** *N. orientalis* **(P)** *N. oxypetala* **(Q)** *N. ciliaris* **(R)** *N. elata* **(S)** *N. damascena* **(T)** *N. stricta* **(U)** *N. d.* ssp. *jenny* **(V)** *N. dengii* ssp. *dengii* **(W)** *N. carpatha* **(X)** *N. a.* spp. *brevifolia* **(Y)** *N. a.* spp. *aristata* **(Z)** *N. a.* spp. *arvensis*; Lateral petals of *Delphinium* and related *Staphisagria* (i-ix) i. *D. anthriscifolium* ii. *S. macrosperma* iii. *D. montanum* iv. *D. bolosii* v. *S. pictum* vi. *D. gracile* vii. *D. verdunense* viii. *D. obcordatum* ix. *D. leroyi*; Dorsal petals in *Delphinium* and its related genera *Staphisagria* and *Aconitum* (x-xvi) x. *D. anthriscifolium* xi. *S. macrosperma* xii. *D. balanse* xiii. *Aconitum* xiv. *D. dasycaulon* xv. *D. sylvaticum* xvi. *D. leroyi*.

Within the genus *Aquilegia*, significant morphological variation is observed. For example, the Asian species *A. ecalcarata* is notable for being spurless and lacking nectaries (**Figure 4A**, Geng et al., 2021), whereas *A. jonesii*, a North American species which is found at high altitudes, lacks the novel organ staminodes (**Figure 4B**, Johns et al., 2024). Variations in petal spur length in *Aquilegia* species are particularly well-studied in North America species, where the spur lengths range from ~5-150 mm (**Figures 5A–K**). This variation in spur length is closely linked to the pollinator specialization, which has played a significant role in the diversification of the genus within the region (Bastida et al., 2010; Puzey et al., 2011).

In addition to spur length, spur shape also varies, with some species exhibiting curved spurs and others straight. For instance, *A. sibirica* (**Figure 5K**) and *A. vulgaris* have curved spurs*, while A. chrysantha* and *A. longissima* exhibit the straight ones (**Figures 5F, H**, Stubben and Milligan, 2007; Puzey et al., 2011; Ballerini et al., 2019). Differences in the shape and length of the blade are also noted (**Figures 5A–K**). The evolution of petal spurs is considered a key innovation within the genus *Aquilegia*, facilitating adaptive radiation by attracting specific pollinators (Hodges and Arnold, 1995; Edwards et al., 2021).

This diversity in morphological characteristics raises numerous questions about the functional relevance and genetic basis of these traits. Particularly intriguing are the genetic and evolutionary mechanisms behind the loss of staminodes in *A. jonesii* and the absence of petal spurs in *A. ecalcarata* (Ballerini et al., 2019; Geng et al., 2021; Johns et al., 2024). Understanding these variations not only sheds light on the evolutionary history of *Aquilegia* but also contributes to broader insights into the processes driving floral diversity and specialization.

### 2.2 Thalictrum

The genus *Thalictrum*, encompassing approximately 150–200 species, exemplifies the complexity and diversity found within the Ranunculaceae family (Boivin, 1944). Distributed throughout temperate regions of the Northern Hemisphere, *Thalictrum* displays a broad spectrum of morphological and ecological adaptations. This genus has experienced considerable diversification, with species adapting to different pollination syndromes and habitats (Kaplan and Mulcahy, 1971).

The divergence of *Thalictrum* from *Aquilegia* is estimated to have occurred approximately 28.7–24.5 Mya, followed by events that contributed to its present widespread distribution (Soza et al., 2013). Notably, *Thalictrum* species exhibit a variety of sexual systems, including hermaphroditism, dioecy (separate male and female plants), andromonoecy (male and hermaphroditic flowers on one plant), gynomonoecy (female and hermaphroditic flowers on one plant) and polygamy with hermaphroditic and unisexual flowers on the same or different individuals (Kaplan and Mulcahy, 1971; LaRue et al., 2013).

The actinomorphic perianth of *Thalicturm* has a calyx composed of deciduous sepals (4-5) that are white or purplish (**Figures 3H–K**). *Thalictrum* lacks petals and nectaries, and the flowers have numerous stamens (34-40) and carpels (4-5) (**Figures 3L, M**, Kaplan and Mulcahy, 1971; Ren et al., 2011). The pistils are unfused and uniovulate, with a stigma that is papillionate (signifying butterfly shape). The flowers produce pollen as the primary attractant for pollinators. Both insect and/or wind pollination have been noted depending on the species (Kaplan and Mulcahy, 1971). For example, *T. thalictroides* is primarily insect-pollinated (entomophilous), *T. polygamum* utilizes both insect and wind pollination (anemophilous), while *T. dioicum* and *T. revolutum* are generally wind-pollinated (Kaplan and Mulcahy, 1971). Insect-pollinated species of *Thalictrum* are characterized by short styles and small anthers, while wind-pollinated species display long styles and large anthers (Martínez-Gómez et al., 2023). This variation in pollination strategies is mirrored in the diverse floral morphologies and sexual systems found within the genus (Kaplan and Mulcahy, 1971).

*Thalictrum* has undergone multiple polyploidy transitions, with chromosome numbers ranging from 2n=2x=14 to 2n=24x=168 (Löve, 1982; Tamura, 1995; Soza et al., 2013). While a study by Soza et al. (2013) found no correlation between changes in ploidy and the evolution of dioecy, they identified a strong association between polyploidy and wind pollination. This diversity in floral structures and pollination strategies not only emphasizes the adaptability of *Thalictrum* species to their environments but also highlights the complex evolutionary processes influencing plant-pollinator interactions. The study suggests that the evolution of dioecy in *Thalictrum* likely involved other pathways beyond polyploidy. Understanding these variations offers valuable insights into the mechanisms driving floral diversity and reproductive success within the genus.

### 2.3 *Nigella damascena* L.

The genus *Nigella* comprises of approximately 18–22 species (Yao et al., 2019; Lubna et al., 2024) These species exhibit an annual life cycle and have a broad biogeographical distribution, native from Macaronesia, Europe to Mediterranean, North Africa, and Central Asia (Kew Science). There is sparse distribution in Oceania region and South America (Nigella L. @ in GBIF Secretariat, 2023). The highest species diversity is found in the Aegean region, where six species of the *N. arevensis* complex are found (Strid, 1970; Bittkau and Comes, 2009; Jaros et al., 2018; Dönmez et al., 2021; Lubna et al., 2024). The genus *Nigella* s. lat. (*Nigella* L. and *Garidella* L.) originated approximately, 43.2-8.9 Mya and has predominantly undergone allopatric speciation. The diversification within the genera approximately began during the Oligocene through the Messinian (29.0-6.0 Mya). The major shift within the genus occurred when the *N. arevensis* complex underwent non-adaptive radiation. The complex probably originated in the Messinian to mid-Pleistocene period (6.2-1.3 Mya) and its diversification began in the Late Pleistocene (0.8-0.2 Mya) (Bittkau and Comes, 2009). This regional event significantly accelerated the diversification rate on the species-level log lineages-through-time (LTT) plot, which is usually linear and constant under the standard constant-rates birth-death (CR-BD) model of diversification. Considering the spatio-temporal scale, it is predicted that the (palaeo) geographical complexity of the Aegean region along with changes in the climate and sea level during the Late Pleistocene are primary factors that led to a rapid increase in speciation within the complex (Bittkau and Comes, 2009). This recent diversification event makes *Nigella* an interesting model system to explore and to further gain insights into the underlying mechanisms that led to such rapid speciation.

The actinomorphic and bipartite perianth of *Nigella* is composed of four kinds of floral organs (Deroin et al., 2015) arranged in an ontogenic spiral (**Figures 3N–P**, Jabbour et al., 2009). The calyx varies in petaloid sepal number (4 –7), with 5 being the most predominant (**Figure 3Q**, Gonçalves et al., 2013; Wang et al., 2015; Zhang et al., 2020). The corolla comprises of bilabiate nectariferous petals (5 - 10) attaching to the receptacle via rod-like stalk (**Figure 3R**, Liao et al., 2020; Zhang et al., 2020). The *Nigella* genus is noted for its elaborated petal morphology (**Figures 5L–Z**). The doubly geniculated petals have a laminar upper lip, bifurcate lower lip covered with short trichomes and long hairs along the pair of pseudonectaries and lobes, respectively (Yao et al., 2019). The true nectary can be found sandwiched between the upper and lower lip of the petal (Liao et al., 2020). The sepals and petals surround the androecium made of stamens (20-25) organized in eight parastichies (**Figure 3S**) and the gynoecium is composed five carpels (**Figure 3T**, Gonçalves et al., 2013; Deroin et al., 2015). *Nigella* also has an apetalous [T] morph, which lacks petals and instead, possesses sepal-like organs. Other floral organs such as sepals, stamens and carpels are similar in structure and cellular composition, however sepal-like organs can vary in shapes ranging from petaloid to bifid or trifid apices to mixed organs with sepal and stamen characteristics (Gonçalves et al., 2013). The pollinators of *N. damascena* include honeybees, which are the most common followed by bumblebees and wasps (Liao et al., 2020).

### 2.4 Delphinium

The species-rich genus was comprehensively reviewed by Sharma et al. (2024); therefore, we provide a brief overview here to maintain context. The highly diverse Delphinieae tribe, comprising approximately 750 species, is characterized by complex zygomorphic flowers that achieve almost perfect monosymmetry through dorsoventral differentiation, with floral organs arranged in a spiral phyllotaxy (Endress, 1999; Jabbour and Renner, 2012a; Novikoff and Jabbour, 2014).

This tribe includes *Delphinium* (~365 species) (Yin et al., 2020) along with the three genera *Aconitum* (~400, species) (Ali et al., 2023), *Gymnaconitum* (1 species) (Wang et al., 2013), and *Staphisagria* (3 species) (Jabbour and Renner, 2011). The members of this group mostly inhabit cold and temperate regions across four continents in the Northern Hemisphere (Bosch et al., 2016) as well as high mountains within tropical Africa (Warnock, 1997; Jabbour and Renner, 2012a; DuPasquier et al., 2021). The tribe originated between 29–31.4 Mya (Jabbour and Renner, 2012a; Wang et al., 2016; Xiang et al., 2017) with the genus *Delphinium* originating about 24 Mya in East Asia. It diversified across the Qinghai-Tibetan Plateau (QTP) where the perennial life cycle emerged and eventually diversified into North America ~3 Mya (Jabbour and Renner, 2012a).

The perianth organs of *Delphinium* are very characteristic, forming a spur(s)-in-spur configuration referred to as a hyperorgan (**Figures 3U, V**, Jabbour and Renner, 2012b; Jabbour et al., 2021; Sharma et al., 2024). The outermost perianth whorl of *Delphinium* flower is denoted by petaloid sepals (5) (**Figure 3W**, Kosuge, 1994; Endress, 1995), two of which are lateral, another two are ventral, and a single dorsal spurred (**Figure 3Y**). The second whorl includes lateral non-spurred trichome-rich showy petals (0-2) except for the subgenus *Consolida* (**Figure 3X**) and nectariferous dorsal spurred petals (1-2) that grow within the dorsal sepal spur cavity (**Figure 3Z**, Jabbour et al., 2021) in synorganization (Chen et al., 2018) forming a hyperorgan (Jabbour et al., 2021). Innermost whorls are composed of stamens (13-40) (**Figure 3i**, Novikoff and Jabbour, 2014; Zhang et al., 2022) surrounding a variable number of carpels (1-5) (**Figure 3ii**, Jabbour and Renner, 2012b; Blanché, 1990; Espinosa et al., 2021; Zhang et al., 2022; Sharma et al., 2024). The distinctive floral structures of these plants are not only aesthetically remarkable but also functionally significant in their interactions with pollinators. The most common pollinators of *Delphinium* are bees, although hawkmoths, hummingbirds, flies and wind pollination are also observed (Jabbour and Renner, 2012b). This genus exhibits a variety of floral morphologies that elucidate their evolutionary relationships, reproductive strategies, and ecological adaptations.

### 2.5 *Papaver* and *Eschscholzia*

The Papaveraceae family holds a pivotal position in the phylogeny of flowering plants, as it is sister to the rest of the Ranunculales, which is sister to the rest of the Eudicots (Peng et al., 2023). This family offers significant insights into the evolution of more complex floral organ morphology (Hidalgo and Gleissberg, 2010). Comprising 45 genera and ~ 850 species, 70% of which are regional endemics, this family is predominantly found from sea level to altitudes of ~ 6000m across temperate regions of the Northern Hemisphere. They inhabit forest understories, alpine meadows, tundra, and deserts (Peng et al., 2023). The Papaveraceae family ancestral habitat is inferred to be a forest understory with partial canopy in Asia (Peng et al., 2023). A divergence of the family occurred during the Lower-Mid Cretaceous resulting in four subfamilies, Hypecoideae, Pteridophylloideae, Fumarioideae, and Papaveroideae (Hoot et al., 2015; Becker et al., 2023; Peng et al., 2024).

The Papaveroideae subfamily originated in Asia. The interconnectivity of western North America via the Bering Land Bridge is hypothesized to be a facilitator for the subsequent dispersal into western North America twice, between 121-101 Mya and 95- 82 Mya (Peng et al., 2023). The Papaveroideae tribe Eschscholzieae includes *Eschscholzia californica* ssp. *californica* (California poppy) (**Figures 3iii–v**), and Papavereae tribe includes *Papaver somniferum* (opium poppy) (Hoot et al., 2015). The genera *Eschscholzia* and *Papaver* serve as model systems for understanding the genetic and evolutionary mechanisms underlying floral development (Hidalgo and Gleissberg, 2010; Becker et al., 2023).

The globally identifiable species within this family is *P. somniferum*, commonly known as the opium poppy. It is native to Northern African countries— Morrocco, Algeria, and Tunisia, and European countries— Portugal, Spain, France and Italy (Jesus et al., 2021). *P. somniferum* is uniquely situated as a close sister group to the core eudicots, making it a prime candidate for comparative studies (Drea et al., 2007; Hong et al., 2022). The flowers of *P. somniferum* are actinomorphic and contain 4 types of floral organs (Hidalgo and Gleissberg, 2010), including sepals (2), followed by two alternating whorls of petals (4, 2 in each whorl) (Hidalgo and Gleissberg, 2010). The next whorl contains numerous stamens (25–223) and carpels (8-12) that fuse to form the capsule (Prajapati et al., 2001; Drea et al., 2007).

*E. californica* also has an actinomorphic perianth, composed of fused sepals (2) arranged in a cap-like structure in the outermost whorl (**Figure 3vi**). The cap dehisces to reveal the brightly colored silky petals (4) that are arranged in two concentric whorls where two outer petals surround two inner petals perpendicularly (**Figure 3vii**, Becker et al., 2005). The color of petals can vary across natural populations. Moving from northern to southern populations, hues will shift from more yellow in the north to orange in central and southern populations (Cook, 1962). Inner to petal whorls are stamens (16–39) that have golden yellow anthers that darken at the base, they attach to the receptacle by a short filament (**Figure 3viii**, Cook, 1962; Becker et al., 2023). The innermost whorl comprises of bicarpellate gynoecium (**Figure 3ix**, Becker et al., 2005, 2023). From the gynoecium, long stigmatic extensions (Becker et al., 2005) protrude in a prong-like fashion.

## 3 Morphological and evolutionary innovations in petal structure and implications for plant-pollinator interactions

**Figure 6:**
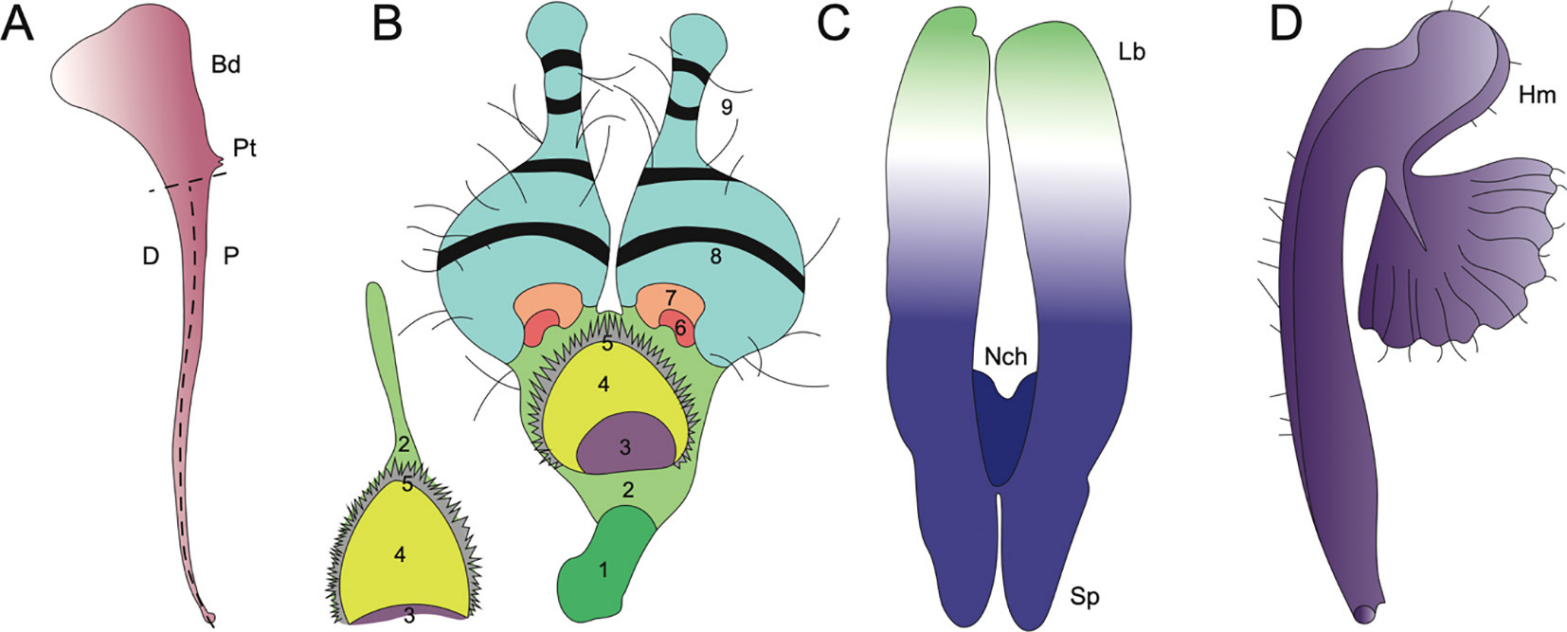
Evolutionary innovations in petal structure for *Aquilegia*, *Nigella*, and *Delphinieae*. (Adapted from Puzey et al., 2011; Yao et al., 2019; Zalko et al., 2021; MNHN and Chagnoux, 2024) **(A)** *A. coerulea* spurred petal distal (D), proximal (P), point of attachment (Pt), blade (Bd) adapted from Puzey et al., 2011. **(B)** *N. arvensis* and its epidermal cell zones 1-9. (1) rectangular cells, (2) irregular cells, (3) secretory, (4) oblong cells, (5) short trichomes, (6) polygonal cells with grainy surfaces, (7) polygonal cells with smooth surfaces, (8) conical cells, (9) long trichomes, and (10) pavement cells (not shown). Adapted from Yao et al., 2019. **(C)** *Staphisagria* notch (Nch), spur (Sp), labium (Lb). Adapted from Zalko et al., 2021. **(D)** *Gymnoaconitum gynandrum* (Hm) helmet adapted from MNHN and Chagnoux, 2024.

Petals can be simple, characterized by flat surfaces, smooth margins, and simple color patterns, as seen in *Arabidopsis*, roses, lilies, and tulips. However, petals can also be elaborate, featuring protrusions, spurs, appendages, trichomes, pouches, fringes, and teeth (Endress and Matthews, 2006; Fu et al., 2022). Such elaborations are prominent in many members of the Ranunculaceae family, including *Aquilegia*, *Delphinium*, and *Nigella* (**Figure 5**). Petal elaboration is observed in at least 35 orders of angiosperms (Fu et al., 2022). This elaboration on petaloid organs enhances plant-pollinator interactions, thereby contributing to reproductive success and promoting evolutionary radiation (Whittall and Hodges, 2007; Yao et al., 2019; Fu et al., 2022). Petal elaborations have been classified into four main types: marginal, ventral, dorsal, and surface elaborations (Endress and Matthews, 2006; Fu et al., 2022). Petal elaborations are generally achieved through morphogenetic transformation which includes deformation and the addition of decorations and details such as those described above to the petal surface (**Figure 6**, Endress and Mathews, 2006; Fu et al., 2022);.

A study by Carrive et al. (2020) cautiously reported insights into the ancestral state of Ranunculales flowers. This study proposed that the ancestral flower was actinomorphic, with trimerous whorls and a differentiated perianth. The study also noted the nectar-storing organs had a single origin. In Ranunculaceae, the nectar is produced and stored by the petals. The ancestral state of petals in the family is characterized as flat petals with short claws, suggesting that the elaboration in petals in various taxa within this family have evolved independently (Delpeuch et al., 2022). Endress and Matthews (2006) mention that within the core Ranunculales, which includes four families, Ranunculaceae, Berberidaceae, Menispermaceae, and Lardizabalaceae, the elaborate and complicated petal morphology is associated with the presence of nectaries in petals. Petal elaboration in Ranunculales, particularly within genera such as *Aquilegia*, *Nigella*, *and Delphinium*, showcases the remarkable morphological diversity and complexity driven by developmental repatterning, heterochrony, genetic regulation and by co-option of existing genetic programs (**Figure 6**, Yao et al., 2019). The study of these complex interactions within an eco-evo-devo framework highlights how ecological interactions, evolutionary processes, and developmental mechanisms converge to shape the elaborate and complex petal morphologies observed in these genera.

### 3.1 The spurred and nectariferous petals of *Aquilegia*

*Aquilegia*, commonly known as columbines, are cherished as ornamentals for their uniquely beautiful flowers, highlighted by petaloid perianth organs and particularly their spurred petals. As mentioned above, the length of these spurs can vary from as short as ~5 mm to as long as 180 mm reflecting the adaptation to different pollinator species with varying tongue lengths (**Figures 5A–K**). The shortest spurs are typically associated with bee pollination, while medium-length spurs are adapted for hummingbirds, and the longest spurs are designed for pollination by hawkmoths (Bastida et al., 2010; Puzey et al., 2011). The elaborate spurs consist of two parts - a laminar blade that grows and extends upwards from its point of attachment to the receptacle, and the spur with the nectary at its tip growing downwards (**Figure 6A**, Edwards et al., 2022). In *Aquilegia*, bee-pollinated species, such as *A. brevistyla, A. jonesii, A. saximontana*, have purple flowers with short, curved spurs. This curvature results from differential cell division and elongation on the distal versus proximal sides of the spur (**Figures 5A, B**, Edwards et al., 2022).

The North American radiation event, approximately 1-3 Mya, resulted in shifts in pollinators, including hummingbirds and hawkmoths (Bastida et al., 2010). These shifts were associated with distinct morphological traits compared to bee-pollinated species. For instance, hummingbird-pollinated species exhibit reduced blade length, red perianths, and long, straight spurs (example, *A. canadensis, A. formosa)* (**Figures 5C, D**, Hodges et al., 2004; Edwards et al., 2022). Notably, there have been two independent transitions to hummingbird pollination. In contrast, hawkmoth-pollinated species also have long, straight spurs but have lost anthocyanins and floral scent (*A*.*longissma, A. pubescens)*, with five documented transitions to hawkmoth pollination (**Figure 5H**, Hodges et al., 2004; Stubben and Milligan, 2007).

Whittall and Hodges (2007) propose that the absence of the hawkmoth pollination syndrome in Europe, despite the presence of hawkmoths, can be attributed to the intermediate role of hummingbird pollination. The tongue length of hummingbirds is intermediate between that of bees and hawkmoths, facilitating a gradual evolutionary transition (Whittall and Hodges, 2007). Spurs are lost in *A. ecalcarata*, a species that is native from central China, and is pollinated by syrphid flies (**Figure 5I**, Huang et al., 2018; Geng et al., 2021). Although the spurless trait in *A. ecalcarata* appears to have evolved from a single evolutionary event, the species itself shows evidence of multiple independent origins, as demonstrated by several studies (Huang et al., 2018; Xue et al., 2020; Geng et al., 2021; Geng et al., 2024). Moreover, recent findings by Geng et al. (2024) present strong evidence that gene flow has played a crucial role in the parallel evolution observed within *A. ecalcarata*. The comparison of gene expression patterns between spurred *Aquilegia* species by Ballerini et al. (2019) and the spurless *A. ecalcarata* reveals a significant heterochronic shift in petal development (Geng et al., 2021). In spurred species, the transition from cell division to differentiation is delayed; this developmental delay allows for the extended formation of the spur (Puzey et al., 2011). The complex relationship between the floral morphology of *Aquilegia* and its diverse pollinators underscores the evolutionary adaptability of this genus. The variations in petal spur length and curvature reflect not only the specialized pollination mechanisms but also the dynamic evolutionary history shaped by geographical and ecological factors.

### 3.2 Distinctly elaborate and complex petals of *Nigella*

The genus *Nigella* is noted for its elaborate petal morphology, showcasing significant variation in micromorphological characters of petals such as upper and bifurcated lower lips, pseudonectaries, nectary chamber, long hairs, short trichomes, and eyebrow-like stripes, etc (Yao et al., 2019; Strid, 1970; Tamura, 1995; Endress and Matthews, 2006; Zhao et al., 2011; Gonçalves et al., 2013). The complex and elaborate petal formation occurred via the deformation of old characters as well as *de novo* formation of new characters (Yao et al., 2019). The developmental basis of petal elaboration in *Nigella* involves both marginal and ventral elaboration. Marginal elaboration is evident from the presence of lobes along the petal edges, while ventral elaboration includes structures such as scales, protrusions, and the development of features like lips and appendages (Endress and Matthews, 2006; Erbar et al., 1999; Yao et al., 2019).

The study by Yao et al. (2019) explores the morphological, genetic, and evolutionary aspects of petal elaboration in *Nigella*, highlighting its complexity and adaptive significance. The evolution of *Nigella* petals exemplifies evolutionary gradualism, where initially simple ancestral petals gradually developed new characters such as long stalks, geniculate bends, and eyebrow-like stripes over time, leading to increased complexity (**Figures 5L–Z**, Yao et al., 2019). The study also highlights the presence of at least ten different types of epidermal cells that contribute to the detailed petal structures in *N. arvensis*. These include rectangular cells, irregular cells, secretory cells, oblong cells, short trichomes, polygonal cells with both grainy and smooth surfaces, conical cells, long trichomes, and pavement cells (**Figure 6B**). Each type of cell has unique micromorphological characteristics, locations, and roles, which collectively enhance the complexity and functionality of the petals (Yao et al., 2019).

In their 2019 study, Yao et al. grouped *Nigella* species into three major categories based on petal morphology and micromorphological characteristics. The ancestral *Nigella* species*, N. integrifolia* is the single species in category 1 (**Figure 5L**). The petals in this category are relatively simple and tubular in structure. Key features include the presence of an upper lip, nectary and ridges, a bifurcate lower lip, and specific epidermal cell types (2,3, 4, 9, and 10). These features represent the ancestral state from which more complex structures evolved. Category 2 includes two species - N*. unguicularis* and *N. nigellastrum* (**Figures 5M, N**). The petals in these species exhibit more complexity compared to Category 1, featuring elongated stalks and geniculate bends, eyebrow-like stripes, and short trichomes. In addition to the cell types in category 1, cell types 1 and 5 are also observed in category 2. N*. nigellastrum* also displays type 11 epidermal cells. Interestingly, type 11 epidermal cells are the characteristic feature of only this species and are not found in other species within the genus. The introduction of these characters increased complexity in the petal structure, creating a clear distinction between the upper and lower lips, as well as the stalk. Consequently, the nectar-bearing petals were directly exposed to pollinators (Yao et al., 2019). These new characters mark an intermediate evolutionary stage, leading to enhanced functionality in pollinator attraction.

Continued evolutionary changes led to the formation of Category 3, which includes all the remaining species within the genus *Nigella*. The species in category 3, exhibit the most elaborate petal structures with highly specialized modifications. In this category, species such as *N. orientalis*, *N. oxypetala*, *N. ciliaris*, *N. elata, and N. damascena* exhibit additional new characteristics such as pseudonectaries, type 7 and type 8 epidermal cells (**Figures 5O–S**). Notably, *N. ciliaris*, *N. oxypetala*, and *N. damascena* independently lost eyebrow-like stripes. Furthermore, epidermal cell type 6 emerged in all species of category 3 except in *N. ciliaris*, *N. oxypetala, N. orientalis.* Besides, caudate lobes are observed in all catagory 3 species except in *N. ciliaris*, *N. orientalis, N. elata* and *N. damascena* Interestingly, pseudonectaries were secondarily lost in *N. degenii ssp. jenny*, and in some populations of *N. degenii subspecies. degenii* and *N. degenii subspecies. barbro* (**Figures 5U, V**).

Within *Nigella*, the origination of the new characters in the petal structure has led to significant functional innovation and complexity. One of the distinguishing features of *Nigella* is the pseudonectaries, or false nectaries. Studies by Liao et al. (2020) suggest that pseudonectaries may play crucial role in pollination success. When observed under the UV light, pesudonectaries appear shiny and reflective, while all the other floral regions are dark black (Liao et al., 2020). Interestingly, bees can view these pseudonectaries as shiny, however, they observe the other flower parts and petals to be dark green, thus indicating the role of pseudonectaries in attracting the bees. Liao et al. (2020) also reported that flowers without pseudonectaries experience significant reduction in the visiting frequency and probing time of honeybees. Besides, the eyebrow like stripes which look like concentric rings on the open flower can serve as tracks that guide the pollinators. Along with the pseudonectaries, they further help the pollinator to locate entrance to the nectary chamber (Weber, 1995).

Notably, the short trichomes are thought to serve a role of protecting the nectar since the nectar chamber is formed by the upper and lower lips, and the short trichomes are found at the chewing surface of the upper and lower lips. Additionally, they also may play role in reducing the evaporation of water as the flowers usually bloom in hot and dry seasons (Strid, 1970; Zohary, 1983).

### 3.3 The lateral flat and dorsal spurred petal (s) in Delphinieae

**Figure 7:**
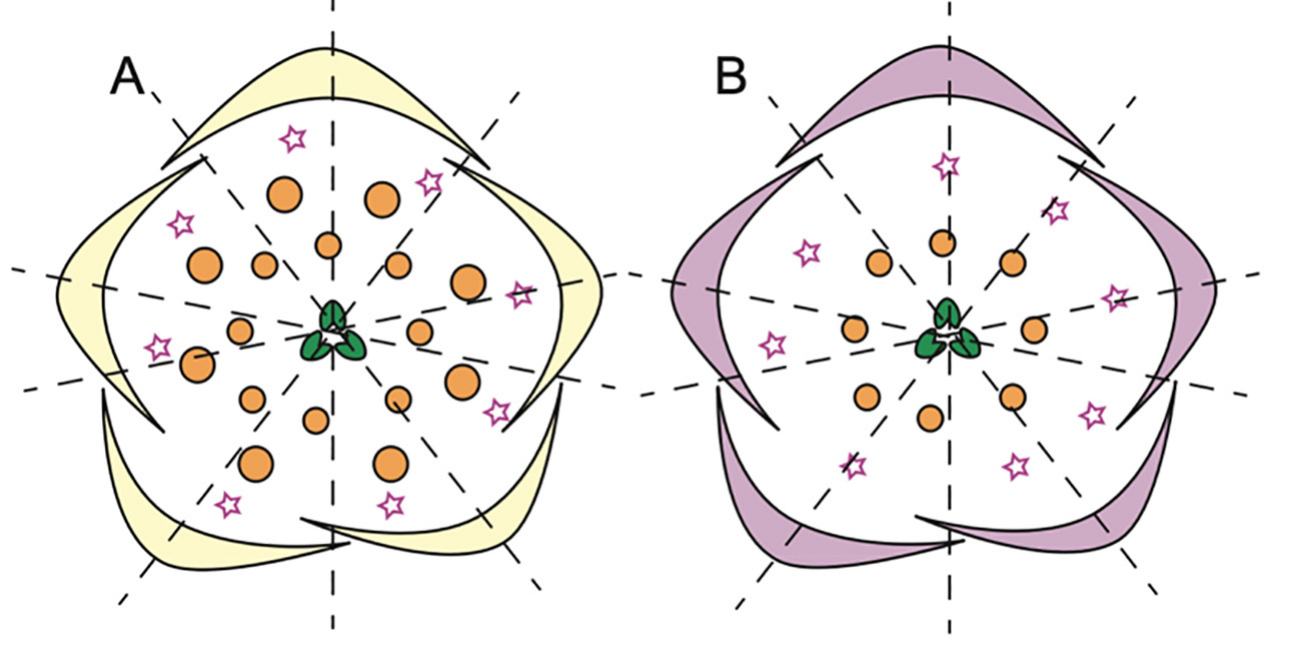
Floral diagrams illustrating species with variations from the typical *Delphinium* perianth structure and actinomorphy. **(A)** *D. turccium* and **(B)** *D. ecalcarata* (Adapted from Espinosa et al., 2017, 2021; Luo et al., 2023), stars in whorl 2 represent reduced petals.

Within the Delphinieae tribe, remarkable diversity in petal morphology is observed including three distinct types of petals–lateral, dorsal- spurred, and reduced ventral. Lateral petals are observed in genera *Staphisagria* and *Delphinium*, except in its subgenus *Consolida* (Jabbour et al., 2021; Zalko et al., 2021). Spurred petals (1–2) are observed in all the species, except in the taxa *D. ecalcarata* and *D. turcicum* (**Figure 7**, Espinosa et al., 2017; Xiang et al., 2017). The reduced petals are present in all species within the tribe, vary in their ranging from 4–6 (Espinosa et al., 2017; Zalko et al., 2021).

The study by Munz (1967) provides a foundational examination of *Delphinium* species, highlighting their unique floral morphologies, including the characteristics of the dorsal spurred and lateral flat petals (**Figures 5i–xvi**). In his meticulous documentation, Munz referred to the spurred petals as “upper pair” with nectariferous spurs and lateral petals as “lower pair” with an expanded lamina and basal claw. These illustrations and descriptions reveal significant diversity in petal morphology across different *Delphinium* species. For instance, the spurred petals, or upper petals, vary in length and curvature while the lower petals, also show considerable diversity in shape and size (Munz, 1967; Jabbour and Renner, 2012b). The variations in petal structure, particularly spur length and shape, are likely adaptations to attract specific pollinators, underscoring the evolutionary significance of these traits in facilitating effective plant-pollinator interactions.

Building on Munz’s observation, the evolution of floral traits within *Delphinium* further illustrates adaptations to various pollinators. For example, the lateral petals in *D. anthriscifolium*, act as visual cues to attract and guide pollinators and function as a biomechanical filter to ensure that effective pollinators access the nectar (Muller, 1883; Blanché, 1990; Hou et al., 2022). Lateral petals are bilobed and twisted helically, providing a stable landing platform for pollinators and partially cover the spurred petals to protect the nectar from desiccation (Zhang et al., 2022; Zhang H. et al., 2024). The dorsal petals possess a nectar spur with a widened tip, designed to accommodate the tongue length of specific pollinators, enhancing pollination specialization (Zhang et al., 2022). Within the tribe, bee-pollinated species typically exhibit shorter, curved spurs, while those pollinated by hummingbirds or hawkmoths tend to have longer, straight spurs (Zalko et al., 2021). In *Delphinium* subgenus *Consolida* flowers possess a single dorsal nectariferous petal, resulting from the fusion of the two superior petals. This structural simplification is seen as an evolutionary adaptation to different ecological niches and pollinator availability. The lateral petals, prominent in the other subgenra of *Delphinium*, are absent in *Consolida*, indicating a trend toward floral simplification (Blanché, 1990).

The *Aconitum* genus showcases a high-galeate upper sepal, forming a hood that protects the nectar-producing parts of the flower (**Figure 5xiii**). The short nectar-spurs of the petals are located at the apex of an elongated pedicel (Jabbour et al., 2021). Bumblebees must enter the hooded sepal to access the nectar spurs, during which their abdomen and legs inevitably brush against the anthers, facilitating pollen transfer (Fukuda et al., 2001; Jabbour and Renner, 2012b). Recently, *Gymnaconitum*, that consists of a single species *G. gymnandrum*, was elevated to the genus level (Wang et al., 2013). This was previously, classified under the genus *Aconitum. G. gymnandrum*, is characterized by several unique features, including clawed sepals, large flabellate petal lobes, multiple carpels and an exposed stigma (**Figure 6D**).

In *Staphisagria*, petal primordia of dorsal petals are fused, the petals become notched and share a common cavity (**Figure 6C**, Payer, 1857; Jabbour and Renner, 2012b; Zalko et al., 2021). Besides, the inner epidermis of inner spur, this shared cavity at the entrance also showcases nectar secretory tissues. The mechanisms of nectar secretion and release exhibit significant variation among species within the tribe (Antoń and Kamińska, 2015; reviewed by Sharma et al., 2024). These varied floral morphologies across the Delphinieae tribe underscore the complex evolutionary dynamics driving adaptation and speciation within the Ranunculaceae family, highlighting the nuanced interplay between floral traits and pollinator interactions.

## 4 Model systems in Ranunculales, key organ identity developments, and potential for studying evolutionary developmental genetics

One of the key strengths of these Ranunculales models is the feasibility of functional genetics studies. Techniques such as RNA interference (RNAi) and Virus-Induced Gene Silencing (VIGS) are applicable across these species, enabling detailed investigations into gene function and regulation. Tissue culture and stable transformation systems are available for *E. californica*, *P. somniferum*, and *T. thalictroides* (Nessler, 1982; Park and Facchini, 2000; Samanani et al., 2002; Chitty et al., 2003; Lotz et al., 2022). While *N. damascena* can be cultured *in vitro*, stable transformation techniques have not yet been established for this species. Notably, the genomes of all these species, except for *Nigella*, have been sequenced or are being assembled. Within the Delphinieae tribe, the genome of *S.picta* is being assembled (The RanOmics group et al., 2024). The continued development of these genetic and genomic resources has facilitated elegant and informative research. Especially, the research on understanding the floral organ identity programs both the conservation and divergence of the ABC model of floral organ identity within closely related species. One particularly intriguing aspect of this research is the role of gene duplication in shaping unique floral morphologies. Below we discuss some selected developments.

### 4.1 Genetic studies in *Aquilegia*

**Figure 8:**
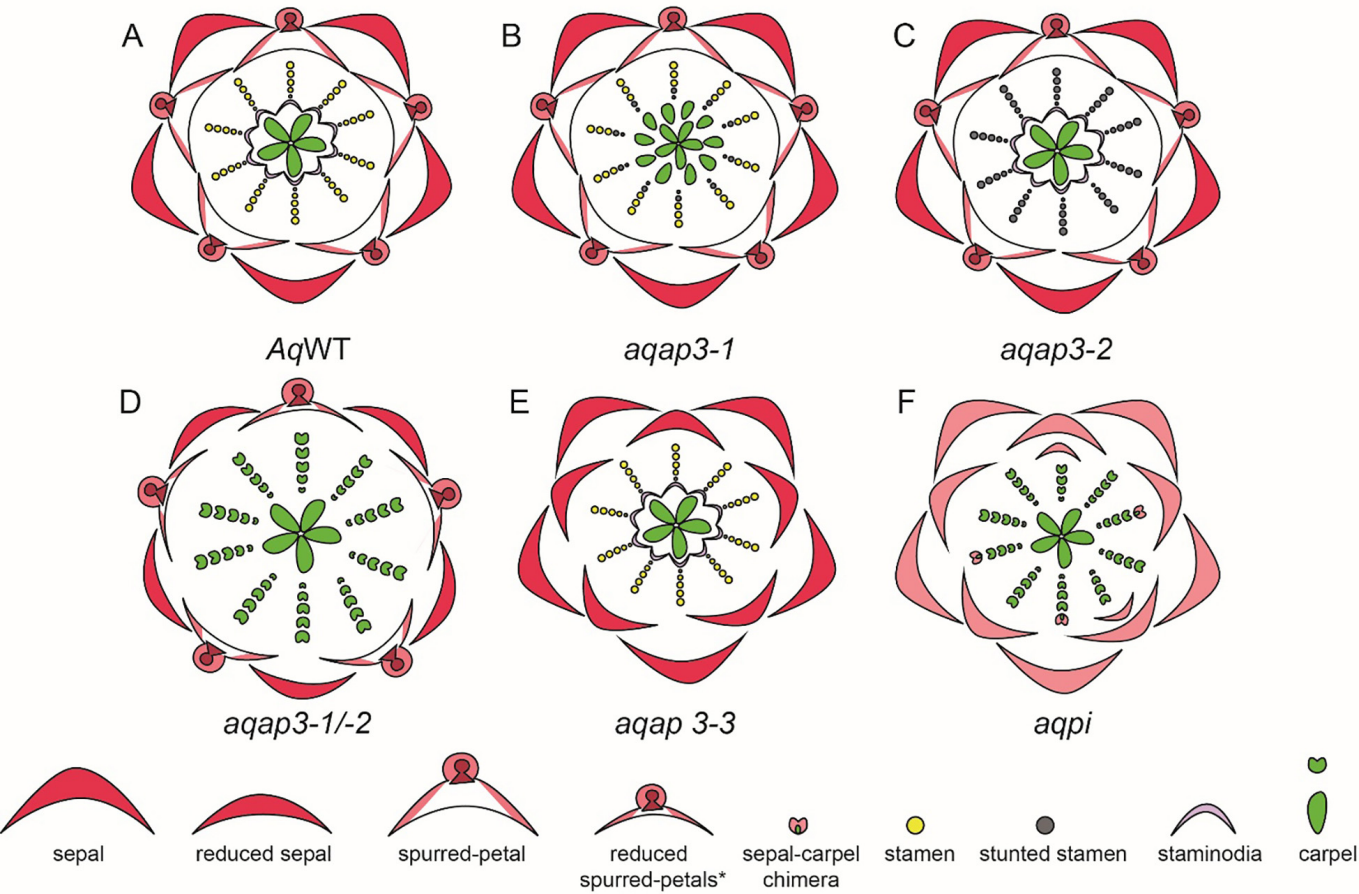
*A. coerulea* and its homeotic RNAi phenotypes (Adapted from Sharma et al., 2014). **(A)** Wildtype (WT) floral diagram displaying five organ identities from outer to inner most whorl: sepals, petals, stamens, staminodia, and carpels. **(B)** *AqAP3-1* silenced flower (*aqap3-1*) show a strong transformation of the staminodia into carpels and stunted, sterile stamens in the inner stamen whorls, indicated in grey. **(C)** *AqAP3-2* silenced flower (*aqap3-2*) display stunted, sterilized stamens in all stamen whorls with no effect on the other floral organs. **(D)** The double knockdown of *AqAP3-1* and *AqAP3- 2* silenced flower (*aqap3-1/2*) exhibits a reduction in petal and sepal size and a full transformation of the staminodia and stamens into carpels. **(E)** In *AqAP3-3* silenced flower (*aqap3-3*) spurred petals are replaced by sepals. **(F)** *AqPI*-silenced flower (*aqpi*) phenotype shows petals replaced by sepals and stamens and staminodia by carpels as well as color loss in sepals and sepal-carpel chimeric organs.

In *Aquilegia*, there are three copies of the B gene homolog *APETALA-3*, *AqAP3-1*, *AqAP3-2*, and *AqAP3-3*. The *AP3* lineage experienced two gene duplication events that occurred before the diversification of Ranunculaceae; these are independent of the B gene duplications in core eudicots. Kramer et al. (2007) hypothesized the role of B genes in contributing to the elaborate petal spur identity and a possible role in establishing staminodium identity. The expression of three *AqAP3* paralogs detected through *in situ* hybridization was discrete in floral meristems at mid-late developmental stages. Expression of *AqAP3-1* was observed only in staminodia, a strong expression of *AqAP3-2* was noted in the stamen whorl and *AqAP3-3* was strongly detected in petal primordia (Kramer et al., 2007), however, a broader expression for *AP3-1*, *AP3-2* was noted in other floral organs in semi quantitative RT-PCR (**Supplementary Table 2.1**). Knockdown studies using the virus-induced gene silencing (VIGS) approach demonstrated the functional role of the three *AP3* paralogs (**Figure 8**). *AqAP3-1* knockdown resulted in the homeotic conversion of staminodia into carpels, inferring the neo-functionalization of this paralog in the establishment of staminodia identity (**Figures 8A, B**). *AqAP3-2* knockdown in severe cases resulted in stamen necrosis but not complete loss of stamen identity (**Figure 8C**). A double knockdown of *AqAP3-1* and *AqAP3-2* resulted in a complete loss of stamen and staminodia identity, resulting in homeotic carpels replacing stamens and staminodes (**Figure 8D**, Sharma and Kramer, 2013). A reduction in petal size was also observed, but the petal identity was not lost. The double knockdown results implicate that *AqAP3-1* to some degree and *AqAP3-2* in a major capacity promote stamen identity. Downregulation of *AqAP3-3* resulted in the loss of petal identity, implicating a petal-specific sub-functionalization (**Figure 8E**, Sharma et al., 2011). Dissection of the roles of the *APETALA*-3 homologs in organ identity establishment in *Aquilegia* is an example of the functional evolution of gene paralogs post duplication.

A single homolog of the *PISTILLATA*, *AqAPI*, was detected in *Aquilegia*. *PISTILLATA* is also a B class gene. *AqPI* is expressed broadly in younger floral meristems but then gets localized in stamen, petal, and staminodia in the late stages (**Supplementary Table 2.1**). The expression pattern of *AqPI*, encompassed the complete expression domain of all three *APETALA 3* paralogs. The *AqPI* expression indicated a conserved canonical role in establishing stamen and petal identity and contributing to staminodia identity; this was confirmed by the initial knockdown studies done in the *Aquilegia vulgaris* (Kramer et al., 2007). Later studies in *A. coerulea* identified a novel role of *AqPI* in the maintenance of the C domain boundary. The *AqPI* knockdown was repeated without the *AqANS* marker; the knockdown not only resulted in the loss of anthocyanin pigment in petals but also a loss of determinacy in the outer stamen whorls (**Figure 8F**). The range of phenotypes included multiple whorls of homeotic sepals or petals instead of stamens, various petal/stamen, or sepal/carpel chimeric organs. These phenotypes indicated that the expression of *AqPI* in outer stamens whorls might be necessary for maintaining the spatial expression domain of *AqAG* homologs (Sharma and Kramer, 2017). Although the role of B genes in the maintenance of petaloidy in sepals was established, we did not observe any phenotype in which the sepal identity was altered.

The A-class gene duplications in core eudicots have resulted in two major lineages, the *AP1* and the *FUL*. Both lineages have some overlap of function, particularly their roles of floral meristem identity, *AP1* also has a role in perianth identity, while *FUL* homologs functions also include inflorescence identity, leaf, and fruit development. Outside the core eudicots, pre-duplication ancestors of *AP1*, and *FUL* gene lineage are the *FUL-like* genes; these genes are more similar to *FUL* than to *AP1* (Litt and Kramer, 2010). In *Aquilegia*, there are two copies of *FUL-like* genes *AqFUL1A* and *AqFUL1B*, the two copies are a result of tandem duplication. Outside the core eudicots, or even outside *Arabidopsis*, there is no conservation of the A class role in promoting sepal identity. In *Aquileia*, *AqFUL* genes are broadly expressed in leaves both pre- and post-vernalization. In young floral meristems, expression can be detected during sepal initiation and in petal, and stamen primordia (**Supplementary Table 2.1**). Knockdown of *AqFUL* results in branched inflorescences with simple cauline leaves coming off laterally from the nodes. The phenotype indicates *AqFUL* has a role in the repression of axillary meristem activity and in leaf morphogenesis (Pabón-Mora et al., 2013). No floral phenotype was observed in the *AqFUL* silenced plants. Functional studies of ABC genes in the non-conventional model system *Aquilegia* have provided valuable insights into the genetic foundations of petal spur development and the genetic mechanisms that define the identity of the novel staminodium.

### 4.2 Genetic bases of organ identity and elaboration beyond ABC genes in *Aquilegia*

Organ identity beyond ABC genes and elaboration mechanism has also been explored in *Aquilegia*. Developmental, transcriptomic, and QTL studies have shed light on the unique characteristics and evolutionary mechanisms of staminodia in *Aquilegia* (Meaders et al., 2020; Johns et al., 2024). Research has demonstrated that staminodia, sterile stamens, evolved approximately 20-22 Mya in the common ancestor of *Aquilegia* and its close relatives, *Semiaquilegia* and *Urophysa* (Bastida et al., 2010; Meaders et al., 2020). Unlike fertile stamens, staminodia lack anthers and feature ruffled lamina that expands to be 2-3 times broader than stamen filaments at maturity. These organs undergo post-genital fusion along their lateral margins to form a protective sheath around the carpels, with lamina expansion identified as a novel developmental mechanism. Asymmetric lignification across adaxial surface is observed in staminodes (Meaders et al., 2020). Key genes related to abaxial/adaxial identity, such as *YABBY* family transcription factors (*AqFIL* and *AqCRC*), were differentially expressed and were upregulated in staminodium compared to stamen, indicating their role in laminar expansion. Besides these, two other genes *HOTHEAD* and *DEFECTIVE IN CUTICLE FORMATION* were enriched in the staminodium implicating their possible role in the adherence mechanism that keeps the 10 staminodium organs attached to each other (Meaders et al., 2020). Within the genus *Aquilegia*, *A. jonesii*, is the only species that lack staminodium. A QTL analysis of an outcrossed F2 population of cross between *A. jonesii*, and *A. coerulea* “Origami” revealed a polygenic basis for staminode loss in *A. jonesii* (Johns et al., 2024).

The elaborate petal spurs in *Aquilegia* bear nectaries at their tips, exhibiting significant variation in length and shape within the genus, with *A. longissima* having the longest spurs (Edwards et al., 2021, 2022). A QTL analysis on the F2 generation from a cross between *A. canadensis* and *A. brevistyla* by Edwards et al. (2021) analyzed 17 floral traits, including spur-related traits such as spur curvature, spur length, and blade length. All traits except spur curvature were controlled by multiple loci (Edwards et al., 2021). In another study, Ballerini et al. (2020) crossed the spurless species *A. ecalcarata* with the spurred species *A. sibirica* and identified a single gene, located under the *POP* QTL and designated as *POPOVICH*, strongly associated with nectar spurs. POPOVICH is a C2H2 zinc-finger transcription factor. Knocking down this gene in *A. coerulea* (the rapid cycling model) resulted in flowers with short or no spurs and without nectaries (Ballerini et al., 2020). Additionally, the role of *TCP4* in petal development was reported by Yant et al. (2015). *TCP4* expression is initially observed in the blade margin of young petals but later shifts to the developing spur. Downregulation of *TCP4* led to spurs with increased curvature and ectopic outgrowths due to cell proliferation. Furthermore, the role of *STYLISH* homologs was also reported in *Aquilegia*. *STY* genes in *Aquilegia* not only have a conserved role in carpel development, but also showed a role in nectary development. Downregulation of all three *STY* homologs resulted in petals without or distorted nectaries (Min et al., 2019).

Building on the insights from the above experiments, Zhang et al. (2020) explored the role of *AUXIN RESPONSE FACTOR* (*ARF*) homologs, *AqARF6* and *AqARF8*, which are highly expressed in developing petal spurs (Yant et al., 2015). The simultaneous knockdown of both *ARF* homologs in *A. coerulea* resulted in a significant reduction in spur length and the absence of nectar production due to the failure of nectaries to mature and become functional. This work provided evidence for the involvement of ARF6/8 homologs in promoting cell expansion and suggested a regulatory connection with *STY* homologs (Zhang et al., 2020). The studies described above contributes to a comprehensive understanding of petal spur development in *Aquilegia.*

### 4.3 Genetic studies in *Thalictrum*

**Figure 9:**
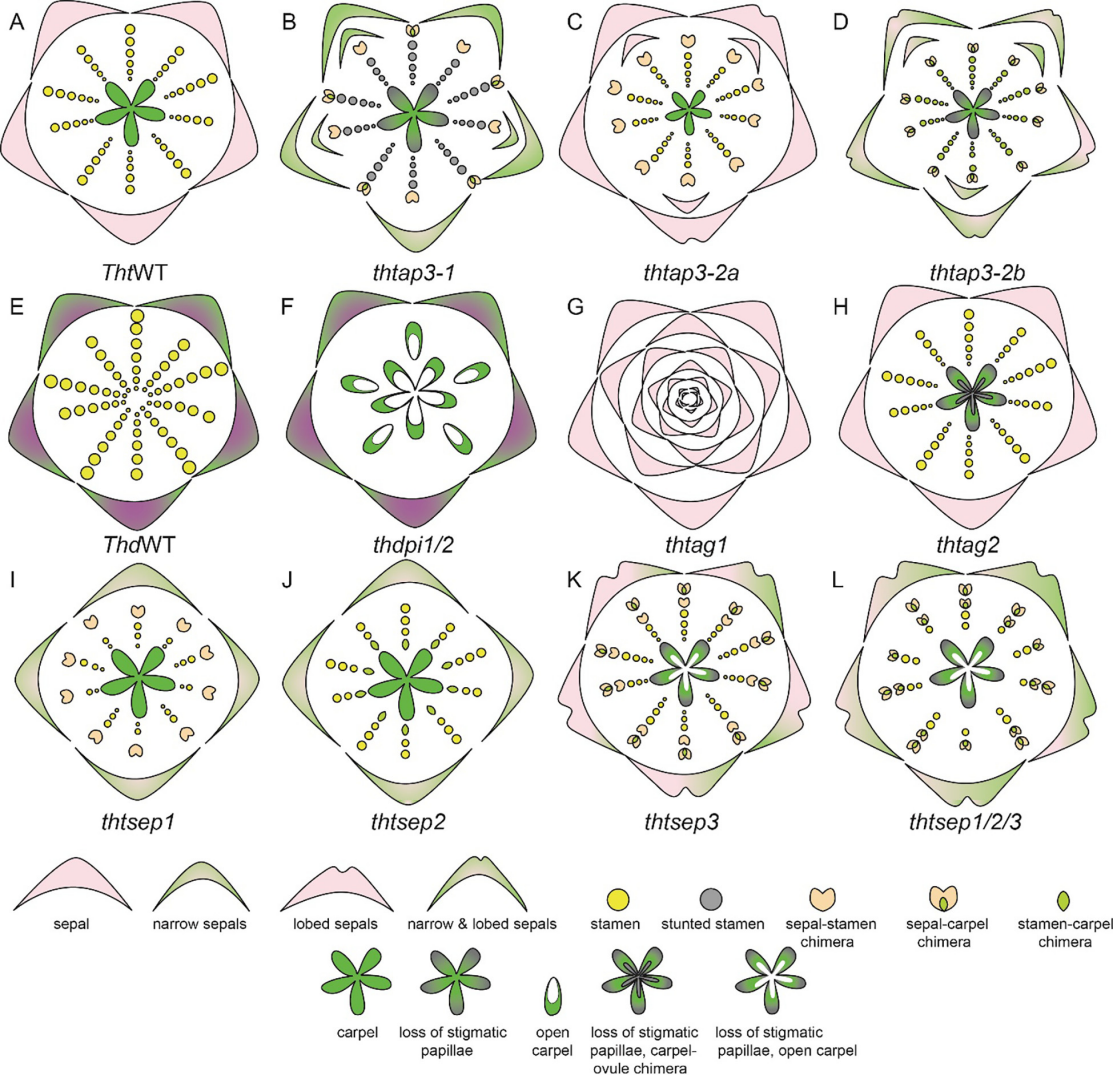
*T. thalictroides* and *T. dioicum* floral diagrams illustrated based on floral phenotype description in Galimba and Di Stilio, 2015; Galimba et al., 2012; LaRue et al., 2013; Di Stilio et al., 2005; Soza et al., 2016; Martínez-Gómez et al., 2020; Galimba et al., 2018. **(A)** Wildtype (WT) floral diagram for *T. thalictroides* displaying three organ identities: sepals, stamen, and carpels **(B)** *thtap3-1* phenotype exhibits a reduction of sepal size and width, extra sepals and chimeric organs in the outer stamen whorls, carpels showing improper stigmatic development **(C)** *thtap3-2a* sepals retain their size but are lobed, extra sepals and chimeric organs observed in the outer stamen whorl **(D)** *thtap3-2b* show a combination of the other two ap3 knockdowns, reduction of sepal size and width, lobed sepals, extra sepals and chimeric organs in stamen whorls a carpels showing improper stigmatic development. **(E)** Wildtype (WT) floral diagram for a male *T. dioicum* male flower with outer whorl sepals and large stamens. **(F)** *thdpi1/2* downregulation produced female flowers in which the stamens are replaced by carpels. **(G)** *thtag1* flowers display stamens and carpels fully transformed into sterile sepal-like organs and floral indeterminacy. **(H)** *thtag2* flowers exhibit ovules transformed into carpel-like structures. **(I)** *thtsep1* displayed reduced or narrowed green-tipped sepals, with modified stamens that are shorter and wider, and sepal-carpel-stamen chimeric organs **(J)** *thtsep2* similar to the phenotype of *thtsep1*, but with carpel-stamen chimeric organs where the inner two whorls meet **(K)** *thtsep3* sepals have irregular edges, green patches and an array of chimeric organs **(L)** *thtsep1/2/3* flowers exhibited a combination of the defects present in the single knockdowns such as patchy sepals, a reduction of stamens, a transformation of stamens to sepaloid stamens, glabrous papillae, and open sterile carpels.

The apetalous genus *Thalictrum* is a very dynamic and elegant model system noted for its diverse sexual system and extensive variability in ploidy. Functional studies conducted using VIGS have unraveled the function of B, C, and E class genes in *T. thalictroides* (**Figure 9**). There are three *AP3* homologs*, AP3-1, AP3-2a, and AP3-2b*, and one or two *PI* homologs depending on the species. The *AP3-3* homolog which has been implicated to have a role in establishment of petal identity (Kramer et al., 2007; Sharma et al., 2011; Zhang et al., 2013) is not detected in *Thalictrum* (Di Stilio et al., 2005). All B class genes are broadly expressed in sepals and stamens (**Supplementary Table 2.2**, Di Stilio et al., 2005). All three *AP3* homologs were silenced individually (Galimba et al., 2018, **Supplementary Table 2.2**). The common knockdown phenotypes of the *AP3* homologs included defects in sepal shape and number and proper establishment of sepal stamen boundary, presence of some chimeric organs such as such as sepal with ectopic anthers or stigmatic tips in stamen whorls and, stunting in stamens showing small anthers and short filaments (**Figures 9A–D**). *TthAP3-1* and *TthAP3-2b* knockdown phenotypes also included reduction in size and width of sepals. Additionally, in both *TthAP3-2a*, and *TthAP3-2b* knockdown phenotypes included lobed sepals (Galimba et al., 2018). These phenotypes indicate a conserved role of B genes in stamen development. Additionally, a role of *AP3-1* and *Ap3-2b* homologs in petaloidy of sepals and proper stigma development in carpels (Galimba et al., 2018).

Expression and functional analysis of *PI* homolog(s) have also been performed in *Thalictrum.* In *T. thalictroides* there is one copy of *PI* homolog that is expressed in sepal and in stamens (Di Stilio et al., 2005; Martínez-Gómez et al., 2020). In the tetraploid*, T. dioicum, a* dioecious species, two transcripts of *PI* homolog have been identified, *ThdPI-1 and ThdPI-2;* both copies are expressed in the perianth organs of carpellate and staminate flowers, as well as in the stamens of staminate flowers (**Supplementary Table 2.2**, Di Stilio et al., 2005; Martínez-Gómez et al., 2020). Downregulation of *ThdPI-1 and ThdPI-2* with one construct that targeted both copies in *T. dioicum* resulted in female flowers in male inflorescences (**Figures 9E, F**, LaRue et al., 2013). In these flowers the stamens were homeotically transformed into carpels. Female plants that were treated with the VIGS did not show any visible phenotype. VIGS treatment was also carried out in *T. thalictroides*, whose flowers are hermaphroditic exhibiting both stamens and carpels. The knockdown flower phenotype was the homeotic conversion of all stamens to carpels. On the basis of these results, LaRue et al. (2013) hypothesized that B genes are a target of mutation, which leads to dioecy in *Thalictrum.*

In *Thalictrum*, the C class expression and functional analysis have yielded very interesting results. In *T. dioicum, ThdAG-1* is expressed in both carpels and stamens whereas *ThdAG-2* is strictly expressed in the carpels of female flowers (Di Stilio et al., 2005). *ThtAG-1* was found to be expressed in stamens, carpels, and ovules, whereas *ThtAG-2* was only slightly expressed in the carpel and ovule (**Supplementary Table 2.2**). Knockdown of *ThtAG1* exhibited homeotic floral phenotypes, including complete conversion of stamens and carpels to sterile organs resembling sepals (**Figure 9G**). Down-regulation of *ThtAG2* resulted in a homeotic conversion of ovules into carpel-like structures (**Figure 9H**, Galimba et al., 2012; Galimba and Di Stilio, 2015). This result indicates a sub-functionalization of *ThtAG2* to an ovule identity gene.

The expression and analysis of E-class gene homologs, *ThtSEP1*, *ThtSEP2*, and *ThtSEP3*, in *T. thalictroides* have been reported (**Supplementary Table 2.2**, Soza et al., 2016; Martínez-Gómez et al., 2020). All three homologs are broadly expressed in all floral organs in both young and mature flowers. However, *ThtSEP3* shows more prominent expression across all floral organs, while *ThtSEP1* is predominantly expressed in the sepals of mature flowers. The knockdown of *ThtSEP1* resulted in flowers with missing and narrowed sepals, and sepals with green tips, short and widened stamens, and some sepal-carpel-stamen chimeric organs (**Figure 9I**, Soza et al., 2016). Knockdown of *ThtSEP2* produced flowers with similar sepal defects, including missing sepals and green patches, as well as fewer stamens, abnormal stamen filaments, and carpel-stamen chimeras at the boundary of the inner two whorls (**Figure 9J**, Soza et al., 2016). The knockdown of *ThtSEP3* led to flowers with irregular sepal edges, green patches, and a range of chimeric organs (**Figure 9K**). Abnormal carpels were observed, including open structures, smooth stigmas lacking papillae, sepaloid stamens, and arrested carpels. Double gene knockdowns of two homologs subsequently such as *ThtSEP1* and *ThtSEP2*, *ThtSEP1* and *ThtSEP3*, and *ThtSEP2* and *ThtSEP3* resulted in enhanced phenotypes, combining the defects seen in single knockdowns (Soza et al., 2016). The triple knockdown of *ThtSEP1*, *ThtSEP2*, and *ThtSEP3* produced flowers with severe defects, including large green patches on sepals, fewer stamens, sepaloid stamens, open and sterile carpels, and smooth stigmas without papillae (**Figure 9L**, Soza et al., 2016). These findings underscore a certain level of redundancy in the functional roles of *SEP* homologs and their role in sepal and stamen development while also contributing to the petaloidy of sepals. Additionally, *SEP3* was implicated in conferring carpel identity, whereas *SEP1* and *SEP2* homologs have a role in maintaining sepal, stamen, and stamen-carpel boundaries (Soza et al., 2016).

### 4.4 Genetic studies in *Nigella damascena*

**Figure 10:**
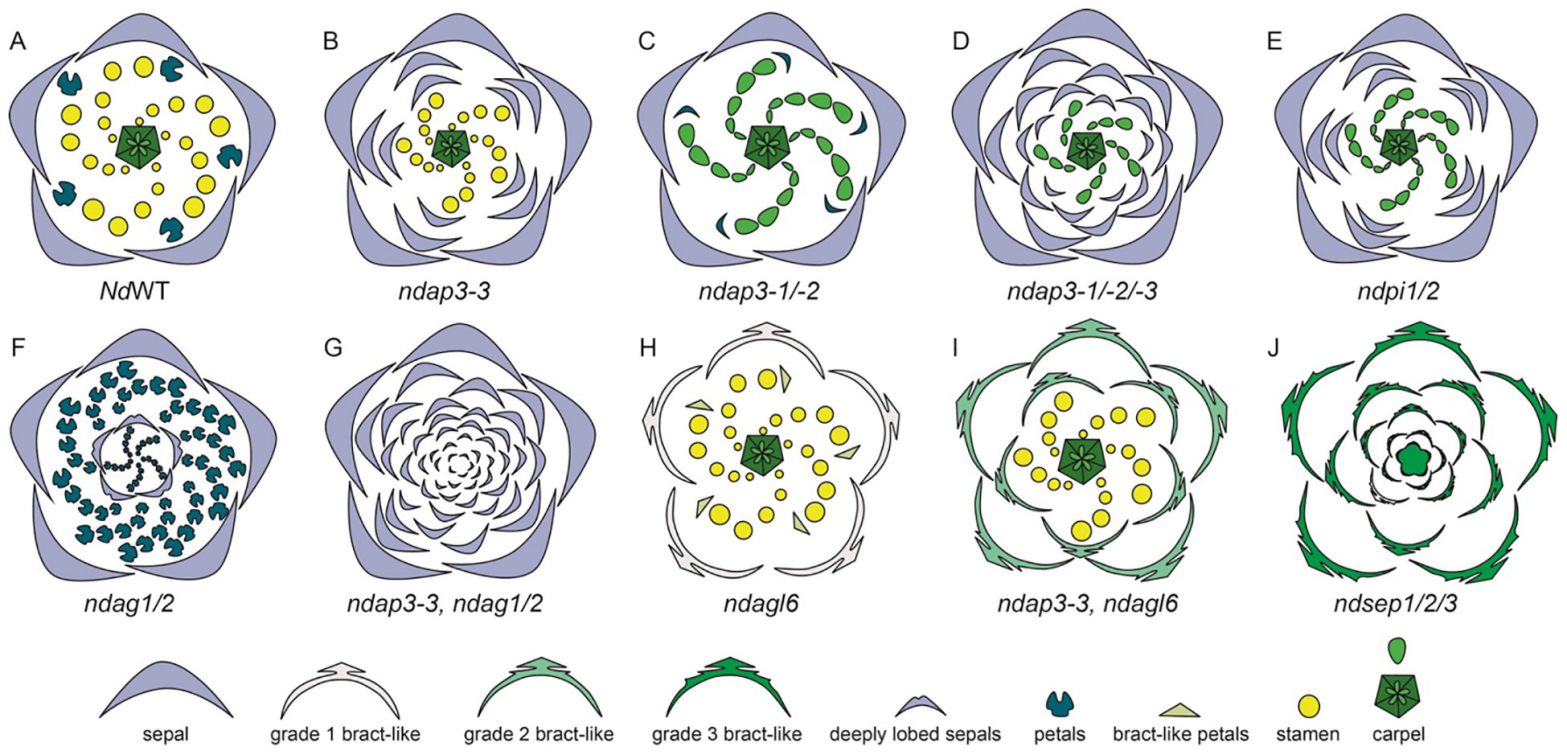
Knockdown phenotypes for wild-type, VIGS-treated, and ‘Double Sepals’ N. *damascena flowers* (Adapted from Gonçalves et al., 2013; Wang et al., 2015). A strong phenotype is shown for each treatment. **(A)** Floral diagram for a wild-type (WT) flower. Gene knockdown performed in the wild type plants: **(B)** *ndap3-3-*silenced flower in which petals are replaced by sepals, extra sepals observed **(C)** *ndap3-1/2*-silenced flower in which petals are reduced, resemble sepals and stamens are replaced by carpels **(D)** *ndap3-1/2/3*-silenced flower in which petals and outer stamens transform into sepals, while inner stamens are replaced by carpels **(E)** *ndpi1/2*-silenced flower in which petals have homeotically transformed into sepals and stamens into carpels **(F)** *ndag1/2*-silenced flower in which “flower-within-flower” structure is observed, where stamens are replaced by petals, carpels replaced by sepals, and floral determinacy is lost. Gene knockdown performed in the ‘Double Sepals’ flower (T morph): **(G)** (T-morph) *ndap3-3, ndag1/2* silenced flower produces unlimited number of sepals **(H)** Knockdown phenotype in the wild type flower for *ndagl6* silenced flower comprises of bract-like sepals with lobed margins, simplified petal structure, and unchanged stamens and carpels and **(I)** T morph, *ndap3-3, ndagl6* silenced flower shows bract-like sepals, unchanged stamens and carpels. **(J)** *ndsep1/2/3* silenced flower shows only bracts, the flower determinacy is lost, and flower inside flower phenotype is observed.

A study by Gonçalves et al. (2013) specifically reported the role of B gene homolog *AP3-3 in N. damascen*a petalous (P) and apetalous (T) morphs. Wang et al. (2015), also in an in-depth study, reported the expression of floral organ function of organ identity genes in *N. damascen*a (**Supplementary Table 2.3**). There are three copies of *APETALA-3*, *NdAP3-1*, *NdAP3-2*, and *NdAP3-3*. *NdAP3-3* is prominently expressed in petals and slightly in stamens. The other two *AP3* homologs, *NdAP3-1* and *NdAP3-2* show broader expression patterns, with stronger expression in the stamens and moderate-to-weak expression in petals and carpels (**Supplementary Table 2.3**). *NdAP3-3* plays a crucial role in the petal identity, and also controls the petal-stamen boundary (Wang et al., 2015). Gonçalves et al. (2013), in their study, show a comparative analysis of B gene expression in the petalous and apetalous morph. Notably, *AP3-3* homolog was not expressed in the T morph while other B genes had similar expression patterns in both morphs (Gonçalves et al., 2013). When *NdAP3-3* was knocked down in petalous morph, flowers with the strongest phenotype produce flowers with ‘double sepals’ as the petals transform into sepals (**Figures 10A, B**). It is interesting to note that the count of sepals was bigger than the sum of petals and sepals observed in the wild-type flowers. This indicated that the petal-stamen boundary was shifted inwards, additionally, *AP3-3* also plays a role in meristem patterning (Gonçalves et al., 2013; Wang et al., 2015).

In their study Gonçalves et al. (2013) suggested that a single mutation event is responsible for the dimorphism, leading to apetalous and petalous flower forms in *N. damascena*. The authors suggest that the insertion of MITE (miniature inverted-repeat transposable element) at the same locus as the *AP3-3* gene has led to the apetalous morphology, the segregation analysis suggest that the same locus is responsible for dimorphism (Gonçalves et al., 2013). The presence of this mutant allele, containing MITE, has been found to be homozygous in the T morph plants, however, it is either heterozygous or absent in the P morph flowers (Zhang et al., 2013). However, other researchers suggest that the MITE insertion is an effect, rather than a cause itself. Interestingly, a recent study by Conde e Silva et al. (2023) confirms the MITE as the major candidate polymorphism that can potentially inactivate *NdAP3-3 in* T morph. There are other changes observed in the T morph, such as the presence of single nucleotide polymorphisms (SNPs), however, none of these were cis-regulatory in nature (Conde e Silva et al., 2023).

When either of the other two *NdAP3* homologs, *NdAP3-1* or *NdAP3-2* is silenced individually, there are no significant phenotypic changes except that the petals are reduced slightly. However, when both *NdAP3-1* and *NdAP3-2* were knocked down simultaneously, the petals resembled sepals which were reduced in size and a stamen to carpel transformation was also observed (**Figure 10C**). This indicated that *NdAP3-1* and *NdAP3-2* together specify the stamen identity, while also controlling the petal development specifically its shape. When all three homologs, *NdAP*3-*1*, *NdAP3-2*, and *NdAP3-3* were knocked down simultaneously, the flowers with strong phenotypes only showed sepals and carpels (**Figure 10D**). Interestingly, the number of sepals was even larger than what was observed in the *AP3-3* knockdown phenotype, implicating that the petal-stamen boundary was further shifted inward. Wang et al. (2015) suggested that *NdAP3-1* and *NdAP3-2* may enhance the function of *NdAP3-3* in determining the petal-stamen boundary.

Furthermore, the expression of *PI-like* genes was also analyzed (**Supplementary Table 2.3**, Wang et al., 2015). Both *PI1* and *PI2* homologs were consistently expressed in sepals, petals, and stamens. When both the *PI* homologs were knocked down simultaneously, the flowers showed only sepals and carpels, implying that *PI-*like and *AP3*-like genes are dependent on each other for proper functionality (**Figure 10E**). However, unlike *AP3*-like genes, In *PI* knockdown flowers the sepal count was not as large as it was observed in flowers when all three *AP3 were* knocked down together.

Additionally, the expression and knockdown studies were conducted on *AG1* and *AG2* homologs (**Supplementary Table 2.3**, Wang et al., 2015). In terms of expression, both the genes, *NdAG1* and *NdAG2*, are expressed in carpels, however, *NdAG1*, is also expressed in stamens. When each of the copies was knocked down individually, no significant phenotype change was observed. However, when both the copies were knocked down simultaneously, a “flower-within-flower” structure was observed with five sepals followed by a precarious number of petals which were a result of stamens being homeotically transformed (**Figure 10F**, Wang et al., 2015). The carpels that homeotically transformed into sepals in the inner whorls were deeply lobed and floral determinacy was lost. Additionally, the petal-stamen boundary was shifted inward as the number of petals was larger than the sum of petals and stamens. Taking all the results into consideration, authors concluded that *NdAG1* seems to play a role as a C-function gene, controlling stamen and carpel identity, and may also be involved in repressing the expression of *NdAP3-3* due to the inward shift of petal-stamen boundary. Additionally, *NdAG2* also specifies carpel identity, plays a role in floral determinacy, and may control the stamen-carpel boundary by potentially regulating B gene expression (**Figure 10G**; Wang et al., 2015). Since single knockdowns for either homolog did not yield any phenotypes, this suggests a redundancy in both homologs’ functional repertoire.

Furthermore, the expression and function of *AP1* homologs, *NdFL1* and *NdFL2*, was determined in *N. damascena* (**Supplementary Table 2.3**). Both homologs were found to be broadly expressed at low levels in all the floral organs. When both the homologs were knocked down simultaneously, no floral phenotype was observed. The expression and function of *NdAGL6* was also studied by Wang et al. (2015) (**Supplementary Table 2.3**). *NdAGL6* is expressed in all floral organs. Knockdown of *NdAGL6* resulted in morphological changes in the sepals, which became bract-like with narrow, linear-lobed margins (**Figure 10H**). Additionally, petals exhibited a linear-to-obovate structure, lacking pseudonectaries, long hairs, and short trichomes, while stamens and carpels remained unchanged. The same phenotype was observed when both *FUL-like* and *AGL6* homologs were knocked down simultaneously, further confirming that *AGL6*, rather than *AP1-*lineage members *NdFL1/2*, plays a crucial role in sepal and petal identity determination.

*NdAGL6* is a dosage-dependent gene that works with *AP3-like* genes to determine basic petal structure (Wang et al., 2015). Wang et al. (2015) also performed VIGS on the T-morph *N. damascena* to further elucidate the function of *AGL6*. In the ‘Double Sepals’ flowers, *ndap3-3-TRV2-NdAGL6*, the resulting floral phenotype included bract-like, pinnately lobed sepals, whose number was higher compared to the *NdAGL6*-silenced flowers, indicating that some of the outer stamens, along with the petals, were converted to bract-like organs (**Figure 10I**). Carpels remained unchanged, demonstrating that *NdAGL6* plays a crucial role in forming margined and petaloid sepals and works with *NdAP3-3* to control petal structure (Wang et al., 2015). The *AGL6* homolog in *N. damascena* is thus implicated in functioning as an A-function gene, determining sepal and petal identity (Wang et al., 2015).

In *N. damascena*, three *SEP-like* genes, *NdSEP1*, *NdSEP2*, and *NdSEP3* have been identified. *NdSEP1* is expressed in the early stages in sepals and petals; *NdSEP2* expression was not detected in any floral organ at any developmental stage (**Supplementary Table 2.3**). *NdSEP3* is broadly expressed in all floral organs across all developmental stages. When all three homologs were knocked down simultaneously, the silenced flowers developed only bracts, and the floral organ determinacy was lost, resulting in a ‘flower-within-flower’ phenotype (**Figure 10J**). Wang et al., 2015 also reported that all *SEP* proteins in *N. damascena* form heterodimers with *NdAGL6*, *NdAG1*, and *NdAG2.* The findings of this study highlight a crucial role of *SEP-like* genes, functioning as E-class genes, in *N. damascena* and having a function in both floral organ identity and ensuring floral organ determinacy (Wang et al., 2015).

### 4.5 Genetic bases of organ identity and elaboration beyond ABC genes in *Nigella*

Liao et al. (2020) performed RNA sequencing to identify candidate genes involved in pseudonectary formation. In their study, they identified *NidaYAB5* as a critical candidate gene. mRNA *in situ* hybridization studies detected the expression of *NidaYAB5* in developing pseudonectaries, with the expression becoming progressively more pronounced at later stages of development. When *NidaYAB5* was downregulated using VIGS, the petals in the silenced flowers did not produce pseudonectaries, confirming the key role of *NidaYAB5* in this process.

Zhang et al. (2020) published a study in which they investigated the function of *NidaLMI1* in relation to petal elaboration. *NidaLMI1* was expressed in petals specifically at developmental stages 8 and 9, in the region between the two pseudonectaries, within the forked lamina, and became more pronounced in areas with short trichomes. Downregulation of *NidaLMI1* resulted in a phenotype where the two petal lobes of the lower lip were fused, and short trichomes were absent. Based on these findings, the authors proposed that *NidaLMI1* plays a crucial role in the differentiation of short trichomes and is essential for the bifurcation of the lower lip.

Together, these findings underscore the complex genetic interactions that drive petal elaboration and organ identity in *N. damascena*.

### 4.6 Genetic studies in *Delphinium*

**Figure 11:**
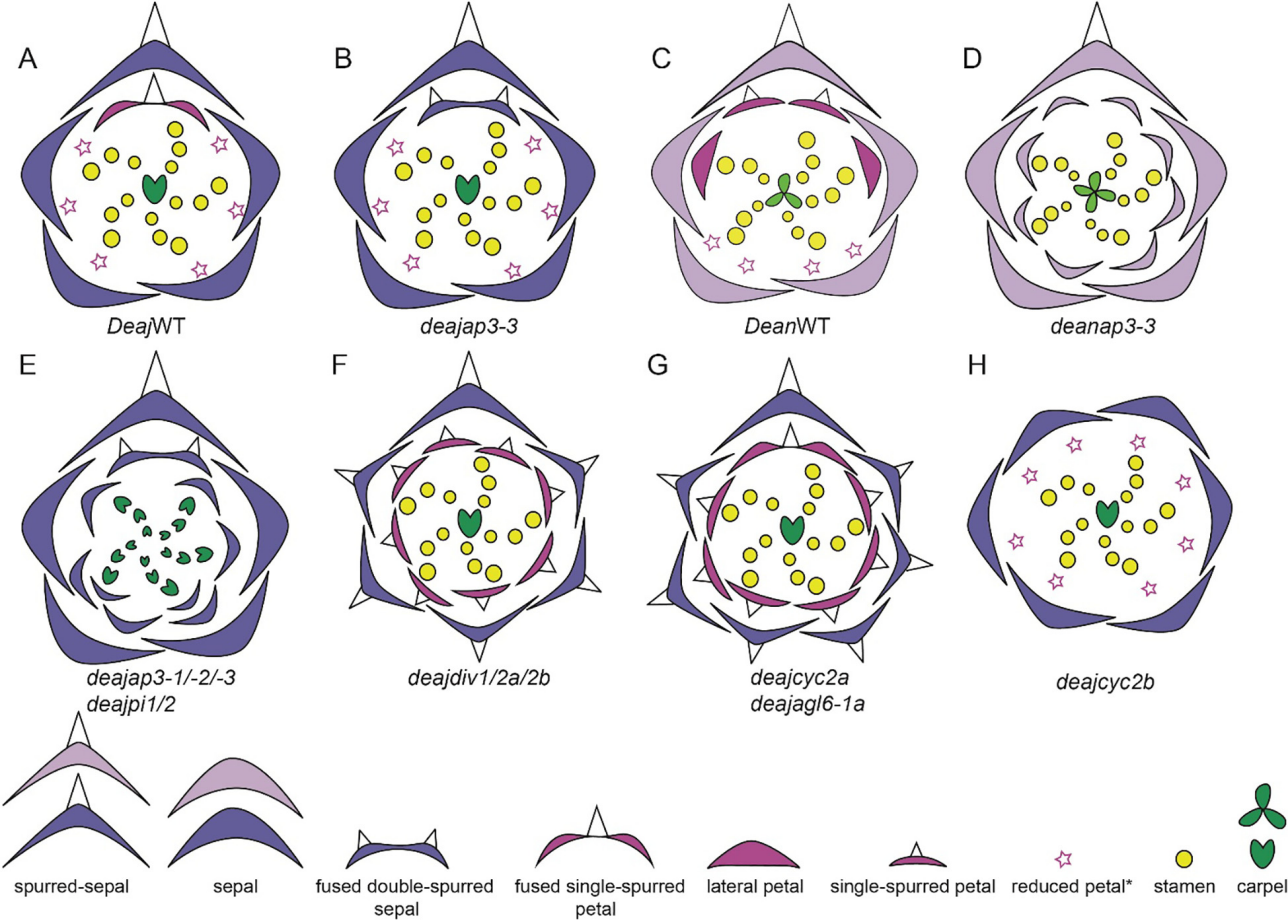
Phenotypes of wild-type, and VIGS-treated *Delphinium* flowers (Adapted from Zhao et al., 2023; Zhang P. et al., 2024). **(A)** Wildtype (WT) floral diagram of *D*. *ajacis*: outer whorl with non-spurred sepals, spurred sepals, the inner whorl single-spurred petal, reduced petals, followed by stamens, and carpels **(B)** *deajap3-3*: fused double-spurred sepals replace single-spurred petal in second whorl, without affecting the floral identity of other organs **(C)** Wildtype (WT) floral diagram of *D. anthricifolium* **(D)** *deanap3-3*: spurred, lateral and reduced petals homeotically transform into sepals in the second whorl **(E)** Phenotype observed when all *AP3-3* homologs are silenced, *deajap3-1*, *deajap3-2*, and *deajap3-3* is similar to when both *PI* homologs are silenced simultaneously: single-spurred petal transforms to fused double-spurred sepals, reduced petals, and some outer stamens to non-spurred sepals, and stamens to carpels. **(F)** *deajdiv1, deajdiv2a*, and *deajdiv2b* triple knockdown: single-spurred sepals replace non-spurred sepals, and all reduced petals and fused single-spurred petals become single-spurred petals. Floral symmetry shifts from zygomorphy to actinomorphy **(G)** Phenotype observed when *deajcyc2a* and *deajajl6-1a* are knocked down individually, the silenced flowers in first whorl have sepals, and all have spurs; in whorl 2 lateral and reduced petals get homeotically transformed into spurred petals; the dorsal fused spurred petal identity is not changed **(H)** *deajcyc2b* silenced flowers: the dorsal spurred sepal in whorl 1 transform into flat sepal; in whorl 2, lateral, dorsal spurred petals become reduced and floral symmetry shifts from zygomorphy to actinomorphy.

The genetic studies in *Delphinium* were reviewed by Sharma et al. (2024), hence we briefly provide some of the major findings in this section, to maintain the necessary context. In *Delphinium*, the B genes *AP3* has three paralogs, *DeajAP3-1*, *DeajAP3-2*, and *DeajAP3-3* and two *PI* homologs *DeajPI1* and *DeajPI2* (**Supplementary Table 2.4**). Functional study by Zhao et al. (2023), in *D. ajacis* implicate a redundant role of *AP3-1* and *AP3-3* homologs in determining reduced petal identity and in maintaining petal stamen boundary. Gene knockdown of *AP3-3* homolog was reported in two *Delphinium* species, first, by Zhao et al. (2023), in *D. ajacis* and then by Zhang et al. (2024) in *D. anthriscifolium*, the results were distinct (**Figures 11A–D**). It is important to note. *D. ajacis*, lacks lateral petals and has only one spurred fused dorsal petal, while *D. anthriscifolium* a species has two lateral petals, and two dorsal nectariferous spurred petals. Zhao et al. (2023) implicate *AP3-3* homolog primarily confers dorsal spurred-petal identity while in *D. anthricifolium AP3-3* may control the identity of dorsal, lateral, and reduced petals (Zhang P. et al., 2024). This distinction in phenotype might be due to a stronger knockdown observed in *D. anthricifolium* or, alternatively, in *D. anthriscifolium* the functional repertoire of *AP3-3* homolog is broader. The triple knockdown of *DeajAP3-1, DeajAP3-2*, and *DeajAP3-3* in *D. ajacis* resulted in flowers with the fused single-spurred dorsal petal transformed into a fused double-spurred sepal, and all reduced petals, along with some outer stamens, were converted into non-spurred sepals (**Figure 11E**). Furthermore, the remaining stamens were transformed into carpels.

The simultaneous knockdown of *DeajP11 and DeajPI2* genes resulted in similar phenotypes to those observed when all homologs are *AP3* homologs are simultaneously knocked down (**Figure 11E**). These classic B phenotypes highlight the functional interdependence of AP3 and PI proteins for floral organ identity determination (Zhao et al., 2023).

### 4.7 Genetic bases of organ identity and elaboration beyond ABC genes in *Delphinium*

*Delphinium* flowers are zygomorphic, the study by Zhao et al. (2023) did functional studies to disentangle the role of *CYCLOIDIA* (*CYC*) and *DIVARICATA* (*DIV*) homologs*. The CYC* homolog was implicated to be a dorsal identity gene promoting spur formation in dorsal petal and sepal, alternatively, *CYC2a*, homolog suppresses spur formation in sepals and reduced petals and is implicated to have a role in establishing lateral and ventral identity in perianth organs (Zhao et al., 2023). Additionally, simultaneous knocked down of *DIV* homologs *DeajDIV1*, *DeajDIV2a*, *DeajDIV2b* yielded very similar phenotypes as *DeajCYC2a.* The most intriguing result was *DeajAGL61a* knockdown phenotype was also similar to *DeajCYC2a*, implicating a complex feedback loop between *CYC2a*, *AGL61a* and *DIV* homologs working jointly to regulate the lateral and ventral development of sepals and petals (**Figures 11F–H**).

In *Delphinium*, the lateral petals are composed of two distinct parts, a distal blade and a proximal stalk. Prior to anthesis, the stalk undergoes a 180° twist, a phenomenon known as resupination. Interestingly, at the junction where the stalk meets the blade, the petal exhibits a unique 90° asymmetric bend. This helical rotation, combined with the bending, plays a crucial role in shaping the complex floral morphology of the plant (Zhang et al., 2022; Zhang H. et al., 2024). RNA-sequencing studies conducted on the lateral petals of *D. anthriscifolium* identified a key candidate gene, *DeanLMI1*, a member of the class I HD-Zip family of transcription factors. Zhang et al. (2024) demonstrated that *DeanLMI1* is essential for the development of asymmetric bending in the lateral petals. When *DeanLMI1* expression was knocked down using VIGS, the lateral petals failed to bend and did not cover the reproductive organs, as seen in wild-type flowers. This study highlights the role of *DeanLMI1* in promoting cell expansion in width and influencing the organization of cell wall nanostructures on the adaxial epidermis, which together contribute to the overall three-dimensional petal shape and asymmetric bending (Zhang H. et al., 2024).

### 4.8 Genetic studies in poppy

**Figure 12:**
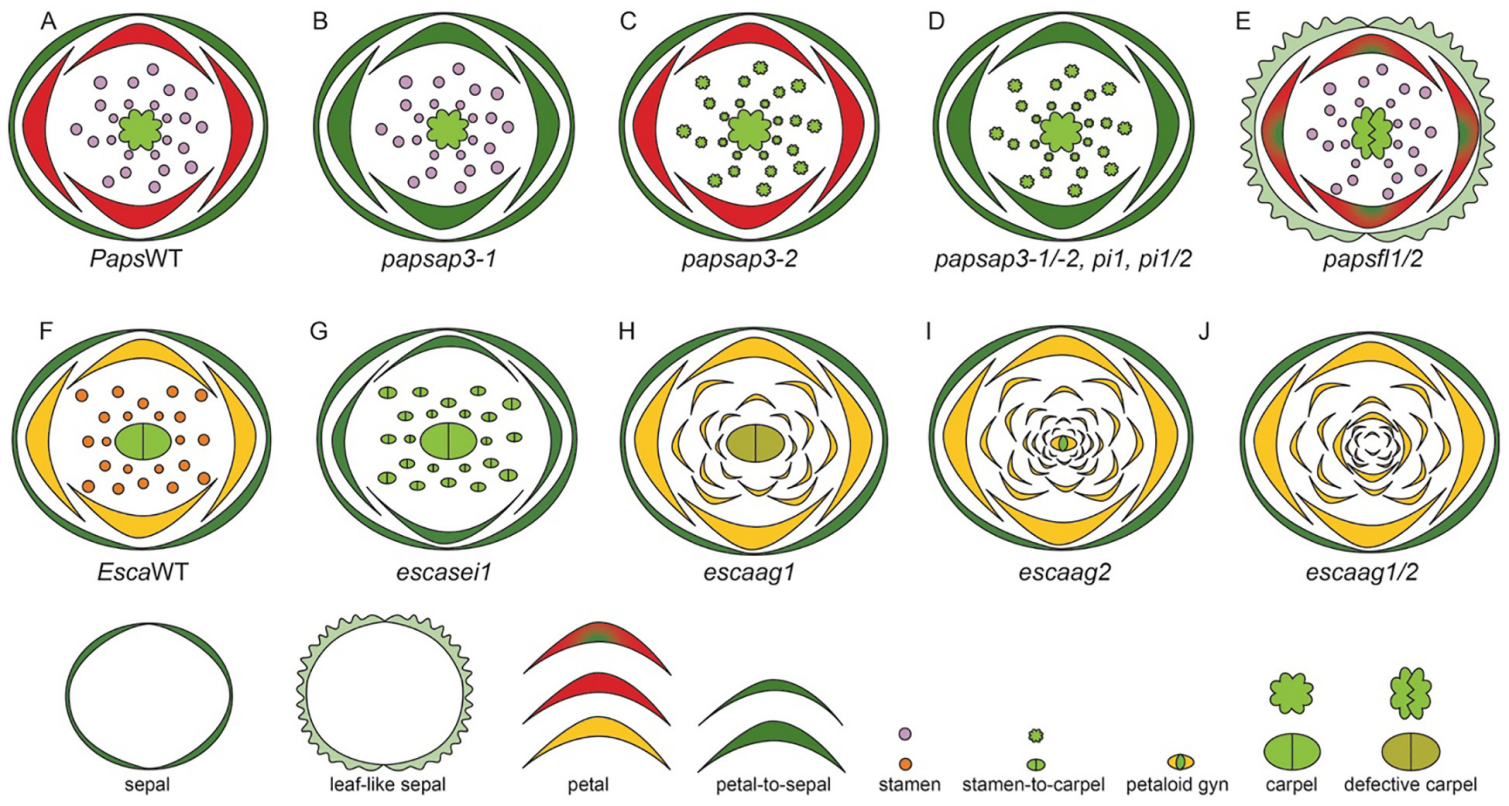
Wild-type and VIGS treated *P. somniferum, and E. californica* flowers. (Adapted from Becker et al., 2023; Hidalgo and Gleissberg, 2010; Pabón-Mora et al., 2012; Lange et al., 2013; Drea et al., 2007; Yellina et al., 2010; Sauquet et al., 2015). **(A)** *P. somniferum*, wildtype (WT) floral diagram displaying five organ identities: sepal cap, petals, stamen, and carpels. **(B)** In *papsap3-1* homeotic transformation of petals to sepaloid organs is observed. **(C)** In *papsap3-2* a stamen-carpel transformation is observed. **(D)** The phenotypes of *papsap3 1* and *2* together, *papspi-1* single gene knockdown and *papspi-1* and *2* together are similar resulting in a classic petal-sepal and stamen- carpel homeotic transformation. **(E)** The *papsfl*-*1* and *2* simultaneous knockdown the phenotype is sepal to leaflike transformation, the petals have green patches, and the fruit is elongated and cracked. **(F)** *E. californica, w*ildtype (WT) floral diagram displaying five organ identities: sepal cap, two petal whorls, several stamen whorls, and bicarpellate gynoecium. **(G)** In *escasei* silenced flowers, petals are transformed into sepals and stamens into carpels. **(H)** In es*ca-ag1* silenced flowers stamens are transformed into petals, petal-stamen mosaic structures, malformed stamens and an undifferentiated gynoecium lacking ovules and placenta. **(I)** In *esca-ag2* silenced flowers stamens and carpels are transformed into petal-like structures. **(J)** In *esca-ag1/2* silenced flowers, stamens transform into petals, and the determinacy is lost with strongly silenced flowers showing flower-within-flower phenotype.

The characterization, expression and functional analysis of ABC genes have been done in *P. somniferum* and *E. californica.* In *P. somniferum*, two *AP3* homologs*, PapsAP3-1* and *PapsAP3-2* were identified*. PapsAP3-1* has a prominent expression in stamens and petals throughout all developmental stages and weak expression in sepals and carpels at later developmental stages (**Supplementary Table 2.5**, Drea et al., 2007). *PapsAP3-2, is* more prominent in stamens at all developmental stages and becomes prominent expression in petals while a weak expression in sepals and carpels at late developmental stages. Silencing of both homologs individually produced distinct phenotypes. *PapsAP3-1* knockdown resulted in the transformation of petals to sepaloid organs with no visible effect on stamens (**Figures 12A, B**). The silencing *PapsAP3-2* yielded stamen-to-carpel transformations (**Figure 12C**). Silencing both the homologs simultaneously resulted in homeotic transformations of petals to sepal and a severe stamen to carpel transformation as compared to single gene knockdown (**Figure 12D**, Drea et al., 2007). These results exemplify sub-functionalization of B gene homologs, with *PapsAP3-1* and *PapsAP3-2* having roles in establishing petal and stamen identities respectively (Drea et al., 2007).

The two *PISTILLATA* homologs *in P. somniferum* are *PapsPI-1* and *PapsPI-2*. *PapsPI-1* is expressed in petals and stamens throughout all developmental stages however, *PapsPI-2* is broadly expressed in all floral organs is only weakly expressed in stamens and carpels at late developmental stages (**Supplementary Table 2.5**). Silencing *PapsPI-1* results in the classic *PI* phenotype yielding homeotic transformation of stamens to carpeloid structures and petals to sepaloid organs. Simultaneous knockdown of both *PI* had a similar phenotype as *PapsPI-1* with more severe stamen to carpel transformation (**Figure 12D**, Drea et al., 2007). These phenotypes exemplify a broader conserved role of *PapsPI-1* in petal and stamen development while a possible redundantly role of *PapsPI-2* with *PapsPI-1* in stamen conferring stamen identity (Drea et al., 2007).

*P. somniferum*, *FUL-like* gene homologs are *PapsFL1* and *PapsFL2* are broadly expressed in all floral organs throughout young and mature developmental stages additionally they are also expressed in leaves and fruits (**Supplementary Table 2.5**, Pabón-Mora et al., 2012). Knockdown studies of *PapsFL1* yielded phenotypes the homeotic transformation of sepal into leaf-like organs that were lobed and had waxy cuticle, the petals had green patches and the most striking results was the asymmetrical elongation and premature rupture of the carpel (Pabón-Mora et al., 2012). The plants that had silenced flowers also had other phenotypes such as branched inflorescences, altered leaf morphology, indicating a conserved function in repressing axillary meristem activity and promoting proper floral development. *PapsFL2* knockdown had very similar phenotypes with the exception that petals were unaffected. Simultaneous knockdown of both copies yielded similar results as seen in both individual knockdowns however, in petals big green patches were observed (**Figure 12E**). These phenotypes implicate that *FUL* like genes in opium poppy fulfill the A function (Pabón-Mora et al., 2012).

Expression and function of *FUL-like* genes was also reported in *E. californica*, *EscaFL1* is broadly expressed in all floral organs except petals, whereas *EscaFL2* is expressed ubiquitously in all (**Supplementary Table 2.5**, Pabón-Mora et al., 2012). Both paralogs are also expressed in leaves and fruits. Knockdown studies indicate that disrupting *EscaFL1-2* results in branched inflorescences with simple cauline leaves, demonstrating their role in repressing axillary meristem activity. The floral phenotype of leaf-like sepals observed in *EscaFL*-silenced plants suggests that the A-class role in promoting sepal identity is conserved in *Eschscholzia californica* as it is in core eudicots (Pabón-Mora et al., 2012).

The *DEFICIENS/APETALA like* homologs in *E. californica* include *EscaDEF1*, *EscaDEF2*, and *EscaDEF3*, which are B-class genes essential for specifying petal and stamen identity. *EScaDEF1* is an ortholog of *APETALA3-2* (*AP3-2*) and is primarily expressed in petals, while *EscaDEF2* and *EscaDEF3* are orthologs of *APETALA3-1 (AP3-1*) and are expressed in stamens and both petals and stamens, respectively (**Supplementary Table 2.5**, Lange et al., 2013). In *E. californica*, *SEI*, was identified as the *PI* homolog. The (VIGS) of the *SEI* gene in *E. californica* resulted in phenotypes that closely resembled those observed in the *sei-1* mutant (Lange et al., 2013; **Figures 12F, G**). Specifically, VIGS-treated plants showed partial homeotic conversion of petals into sepals, where the altered petals retained some orange coloration but exhibited a broad green stripe in the center, making them sepaloid in size and shape. Additionally, the stamens in the outer whorls were transformed into reduced-sized petaloid organs, and in place of the inner whorl stamens, single, unfused carpel-like structures developed. Lange et al. (2013) proposed that B class genes expression possibly have a reinforcing effect on C class genes, specifically in species that have multiple stamen whorls.

In *E*. *californica* there are two *AGAMOUS* homologs, expression and functional analysis has been reported for these. *EscaAG1* highly expressed in stamens and carpels and low expression is observed in the perianth organs (Yellina et al., 2010). *EScaAG2* is carpels and more prominently in stamens (**Supplementary Table 2.5**). The study by Yellina et al. (2010) explores the functional role of the *AGAMOUS* (*AG*) homologs using virus-induced gene silencing (VIGS). Knockdown of *EscaAG1* results in flowers showing transformation of stamens into petal-like organs (**Figure 12H**). Most flowers exhibited partial conversion, affecting only the outer whorl of stamens. The gynoecium also displayed alterations, transforming into flattened green structures or petaloid organs with orange pigmentation, indicating a loss of carpel identity. Similarly, knockdown of *EscaAG2* resulted in homeotic conversion of stamens and carpels. The gynoecium in these cases also exhibited petaloid features, further indicating a loss of carpel identity (**Figure 12I**).

The simultaneous knockdown of both *EscaAG1* and *EscaAG2* produced the strongest phenotypes, of flowers showing homeotic conversions of stamens into petals. Additionally, the gynoecium showed more pronounced petaloid transformations, emphasizing the combined role of both genes in maintaining carpel identity (**Figure 12J**). Beyond changes in organ identity, VIGS treatment also caused defects in floral meristem termination, resulting in increased stamen numbers and prolonged meristematic activity, leading to the development of ectopic floral structures within the gynoecium. These findings highlight the essential roles of *EscaAG1* and *EscaAG2* in specifying stamen and carpel identity, as well as in ensuring proper floral meristem termination in *E. californica* (Yellina et al., 2010).

## 5 Conclusions and synthesis

Ranunculales model systems provide invaluable insights into plant evolution and development, owing to their diverse and distinctive morphological traits and important phylogenetic position. Through the review of eco-evo-devo studies in these model systems it is clear that morphological diversification is shaped by ecological, evolutionary and genetic factors. However, the underlying mechanisms driving this diversification differ significantly among the model systems. The significance of gene duplication is profound, as gene orthologs and paralogs in closely related species show both conserved and diverged functions. For example, in *Aquilegia AP3-1* has been neo-functionalized and establishes the identity of the novel organ staminodia (Sharma and Kramer, 2013), whereas in *Thalictrum AP3-1* homolog controls the size, width, and petaloidy of sepals and proper stigmatic development (Galimba et al., 2018). Furthermore, in *Aquilegia*, *Delphinium* and *Nigella*, *AP3-2* homolog has a role in conferring stamen identity (Sharma and Kramer, 2013; Wang et al., 2015; Zhao et al., 2023), however, in *Thalictrum* it controls sepal shape and petaloidy of sepals (Galimba et al., 2018).

Another example is the case of *Thalictrum*, where *SEP3* homolog has a more pronounced role in carpel identity, however, in *Delphinium* and *Nigella, SEP* genes broadly control organ identity of all floral organs. Another interesting difference lies in the role of *AGL6* in *Delphinium* and *Nigella*. *AGL6* homologs, *AGL6-1a* and *AGL6-1b*, establish spur identity in *Delphinium* and work redundantly to determine sepal and petal identity, however, in *Nigella*, *AGL6* functions as an A class gene playing role in sepal and petal identity (Wang et al., 2015). Furthermore, the gene knockdown studies in *Aquilegia* and *Nigella* suggest negligible role of *FUL-like* genes in floral organ identity, however, in Poppy, *FL1* and *FL2* homologs play role in promoting sepal identity. Another interesting example is the diversification of *AG2* homologs, in *Thalictrum AG2* has been implicated to have a D function conferring proper ovule development.

Research in the recent years have not only enhanced our understanding of floral evolution but it also acknowledges several caveats that must be considered. Below we outline key advantages and realistic caveats associated with for eco-evo-devo research using these model systems.

### 5.1 Advantages

- The model systems described in this study provide unique insights into early-diverging eudicot lineage and the molecular changes level that contributed to rapid angiosperm diversification. For example, *Aquilegia* that has undergone recent adaptive radiation, with interfertile species showing low genetic divergence.
- Availability of flower mutants in *Aquilegia*, *Nigella*, *Delphinium* and *Thalictrum* provides an opportunity for comparative developmental and genetic studies.
- Opportunity to study the morphological evolution of elaborated and novel floral organs in conjunction with their ecological and molecular bases.
- The growing availability of genetics and genomic resources, such as provided by the RanOmics group, enable in-depth omics-studies and accelerates research.

### 5.2 Challenges

- Diversity of habitats and limited availability of some species can constrain comparative studies.
- Some species, like *Delphinium* have large genomes which can make assembly and analysis challenging.
- Rapid transformation and efficient systems are not available for all models (reviewed by The RanOmics group et al., 2024).
- Comparatively longer lifecycles than *Arabidopsis.*
- In some species like *Aquilegia* inbreeding depression can pose challenges in maintaining both pure and mutant lines.
- Unavailability of mutant seed banks limits broad genetic screening studies.

## 6 Future directions

The wealth of floral morphological diversity in early-diverging Ranunculales presents a great opportunity to understand the molecular bases and evolutionary transitions and innovations. Below are a few future directions research in this field.

- Adaptive and non-adaptive radiation within the Ranunculales, play crucial roles in shaping floral diversity. Adaptive radiation, driven by natural selection and pollinator interactions, leads to specialized floral traits that enhance reproductive success in specific ecological niches, as seen in genera like *Aquilegia*. Conversely, non-adaptive radiation, influenced by genetic drift and geographical isolation, contributes to the broad spectrum of floral forms, as exemplified by *Nigella*. Future research should focus on elucidating the genetic and developmental pathways underlying these processes. By integrating genomic and ecological approaches, we can deepen our understanding of how evolutionary forces interact to drive speciation and floral diversification in Ranunculales.
- Studies in *Aquilegia* have demonstrated that many floral traits are polygenic, with QTLs distributed across different chromosomes. This discovery lays a robust groundwork for future investigations into the genetic interactions and regulatory mechanisms underlying these traits. Future studies can build on this foundation to explore the genetic networks that govern floral development and diversification.
- Investigating the genetic and environmental factors driving the evolution of diverse sexual systems within *Thalictrum*, such as hermaphroditism, dioecy, and andromonoecy. Future studies could involve studying the genetic regulatory networks that regulate sex determination and the ecological and evolutionary implications of different sexual systems on reproductive success and fitness.
