# Supplementary material for: Comparative case study of evolutionary insights and floral complexity in key early-diverging eudicot Ranunculales models
Source: Front Plant Sci. 2024 Oct 30;15:1486301. doi: 10.3389/fpls.2024.1486301 (PMC11557424; doi:10.3389/fpls.2024.1486301)
Supplement: Supplementary file 1 [file Table1.docx]

*Supplementary Material*

**Comparative Case Study of Evolutionary Insights and Floral Complexity in Key Early-Diverging Eudicot Ranunculales Models**

*** Correspondence:** Corresponding Author: [bsharma@cpp.edu](mailto:bsharma@cpp.edu)

**Table 1**. Comparative morphological traits among Ranunculales species. Symbols: * tepals, - no data available, † fused, ‡location data obtained from [*Plants of the World Online | Kew Science*](https://powo.science.kew.org/). Abbreviations: Act: Actinomorphic, Zyg: Zygomorphic, P: Perennial, A: Annual, B: Bees, BB: Bumblebees, H: Hummingbird, HM: Hawkmoth, F: Flies, W: Wasps.

| ***Family*** |  | **Morphology** | | | | | | | | | | | | | |
| --- | --- | --- | --- | --- | --- | --- | --- | --- | --- | --- | --- | --- | --- | --- | --- |
|  | Sepal | Sp. Sepal | Petals | Sp. Petal/s | R. Petal | L. Petal | Staminodia | Nectary | No. Stamens | Carpel | Symmetry | Life Cycle | Pollinators | Native Range | Reference |
| ***Aquilegia*** |  |  |  |  |  |  |  |  |  |  |  |  |  |  |  |
| ***coerulea*** | 🗸 |  | 🗸 | 🗸 |  |  | 🗸 | 🗸 | 40-130 | 4-10 | Act | P | B, BB, HM | N. America | 4, 8, 33, 40, 47, 48 |
| ***ecalcarata*** | 🗸 |  | 🗸 |  |  |  | 🗸 |  | 10, 40-60 | 5 | Act | P | F | China | 3, 4, 19, 25, 30, 48, 49 |
| ***formosa*** | 🗸 |  | 🗸 | 🗸 |  |  | 🗸 | 🗸 | 40-70 | 5 | Act | P | H | N. America | 3, 14, 21, 49 |
| ***jonesii*** | 🗸 |  | 🗸 | 🗸 |  |  |  | 🗸 | 40-70 | 4-6 | Act | P | BB | N. America | 21, 23, 30, 33, 48 |
| ***pubescens*** | 🗸 |  | 🗸 | 🗸 |  |  | 🗸 | 🗸 | 40-70 | 4-6 | Act | P | HM | N. America | 22, 49 |
| ***Delphinium*** |  |  |  |  |  |  |  |  |  |  |  |  |  |  |  |
| ***ajacis*** | 🗸 | 🗸 | 🗸 | 🗸 | 🗸 |  |  | 🗸 | 13-17 | 1 | Zyg | A | BB, F | Eurasia^‡^ | 27, 58 |
| ***anthriscifolium*** | 🗸 | 🗸 | 🗸 | 🗸 | 🗸 | 🗸 |  | 🗸 | 12-22 | 3 | Zyg | A | BB | China | 6, 26, 52, 56, 57 |
| ***ecalcaratum*** | 🗸 |  | 🗸 |  | - | - |  | - | 5 | 3 | Act | - | - | China | 15 |
| ***grandiflorum*** | 🗸 | 🗸 | 🗸 | 🗸 | 🗸 | 🗸 |  | 🗸 | - | 3-5 | Zyg | P | BB | Eurasia^‡^ | 15, 28 |
| ***turcicum*** | * |  | * |  | 🗸 |  |  |  | 12-18 | 3 | Act | A | - | C. Turkey | 10, 16, 50 |
| ***Consolida*** |  |  |  |  |  |  |  |  |  |  |  |  |  |  |  |
| ***regalis*** | 🗸 | 🗸 | 🗸 | 🗸 | 🗸 |  |  | 🗸 | 13-20 | 1 | Zyg | A | B, BB | Medit. | 7, 9, 15 |
| ***Aconitum*** |  |  |  |  |  |  |  |  |  |  |  |  |  |  |  |
| ***lycoctonum*** | 🗸 | 🗸 | 🗸 | 🗸 |  |  |  | 🗸 | 17-35 | 3 | Zyg | P | BB | Europe | 1, 2, 24, 27, 32 |
| ***Gymnaconitum*** |  |  |  |  |  |  |  |  |  |  |  |  |  |  |  |
| ***gymnandrum*** | 🗸 |  | 🗸 | 🗸 | 🗸 |  |  | 🗸 | 30-90 | 6-14 | Zyg | A | BB | Qinghai-Tibetan Plateau | 38, 56, 58 |
| ***Staphisagria*** |  |  |  |  |  |  |  |  |  |  |  |  |  |  |  |
| ***macrosperma*** | 🗸 | 🗸 | 🗸 | 🗸 | 🗸 | 🗸 |  | 🗸 | ~40 | 3 | Zyg | A | BB, HM, F | Medit.^‡^ | 27, 55, 58 |
| ***picta*** | 🗸 | 🗸 | 🗸 | 🗸 | 🗸 | 🗸 |  | 🗸 | ~40 | 3 | Zyg | A | BB, HM, F | Medit.^‡^ | 27, 55, 58 |
| ***Thalictrum*** |  |  |  |  |  |  |  |  |  |  |  |  |  |  |  |
| ***dioicum*** | 🗸 |  |  |  |  |  |  |  | 20-42 | 5-17 | Act | P | Wind | N. America | 12, 36 |
| ***thalictroides*** | 🗸 |  |  |  |  |  |  |  | 45-76 | 3-11 | Act | P | B, BB, F | N. America | 18, 31, 39, 42 |
| ***Nigella*** |  |  |  |  |  |  |  |  |  |  |  |  |  |  |  |
| ***damascena*** | 🗸 |  | 🗸 |  |  |  |  | 🗸 | 30-45 | 3-5^†^ | Act | A | B, BB, W | Medit. | 28, 29, 37, 59 |
| ***Papaver*** |  |  |  |  |  |  |  |  |  |  |  |  |  |  |  |
| ***Eschscholzia californica*** | 🗸 |  | 🗸 |  |  |  |  |  | 16-39 | 2^†^ | Act | A | B, BB, Wind | N. America | 5, 11, 20 |
| ***somniferum*** | 🗸 |  | 🗸 |  |  |  |  |  | (25)60-223 | 8-12^†^ | Act | A | B, F | Medit. | 13, 20, 41, 43 |

**Table 2**. Expression of floral organ identity (MADS box) genes in five genera (2.1-2.5) within the Ranunculales order. Plus and minus signs correspond to the presence and absence of expression, respectively. The slash is used to indicate observed expression change as the flower transitions from early to late developmental stage. S, strong expression; w, weak expression; ND, no data available.

**2.1** *Aquilegia coerulea*

| *Aquilegia coerulea* | | | | | | |
| --- | --- | --- | --- | --- | --- | --- |
| Gene | Sepal | Petal | Stamen | Staminodium | Carpel | References |
| *AqvAP3-1* | + (w) | + (w) | + (w) | + (s) | + (w) | 34 |
| *AqvAP3-2* | + (w) | + | + (s) | + (w) | + (w) | 34 |
| *AqvAP3-3* | - | + (s) | + (w) | - | - | 34 |
| *AqvPI* | + (w) | + | + | + (w) | + (w) | 34 |
| *AqcFL1^*^* | + (w) | + | + | + | + | 45 |
| *AqcAG1* | ND | ND | + | ND | + | 34, 46 |
| *AqcAG2* | ND | ND | ND | ND | + | 34, 46 |

******AqcFL1A* and *AqcFL1B*

**2.2** *Thalictrum thalictroides* and *Thalictrum dioicum*

| *Thalictrum thalictroides* and *Thalictrum dioicum* | | | | | |
| --- | --- | --- | --- | --- | --- |
| Gene | Sepal | Stamen | Carpel | Ovule | Reference |
| *ThtAP3-1* | +(s) | + | - | ND | 12, 39 |
| *ThtAP3-2a* | +(w) | + | - | ND | 12, 39 |
| *ThtAP3-2b* | +(s) | + | - | ND | 12, 39 |
| *ThtPI* | +(s) | + | - | ND | 12, 39 |
| *ThtAG1* | - | + | +(w) | +(s) | 17, 39 |
| *ThtAG2* | - | - | +(w) | + | 17, 39 |
| *ThtSEP1* | + | +(w) | - | ND | 39 |
| *ThtSEP2* | + | + | + | ND | 39 |
| *ThtSEP3* | + | + | + | ND | 39 |
| *ThdPI-1* | + | + (Staminate) | - | - | 12 |
| *ThdPI-2* | + | + (Staminate) | - | - | 12 |

**2.3** *Nigella damascena* (Reference:51)

| *Nigella damascena* | | | | |
| --- | --- | --- | --- | --- |
| Gene | Sepal | Petal | Stamen | Carpel |
| *NdAP3-1* | - | + | + (s) | +/- |
| *NdAP3-2* | -/+ | +/- | -/+ | +/- |
| *NdAP3-3* | - | + (s) | +/- | - |
| *NdPI1* | + | + (s) | + (s) | +/- |
| *NdPI2* | + (s) | + (s) | + (s) | +/- |
| *NdFL1* | + | +/- | +/- | + |
| *NdAG1* | - | - | -/+ | + |
| *NdAG2* | - | - | - | + |
| *NdSEP1* | +/- | +/- | +/- | +/- |
| *NdSEP3* | + | + | + | + |
| *NdAGL6* | + (s) | + (s) | + | + |

**2.4** *Delphinium ajacis* (Reference:58)

| *Delphinium ajacis* | | | | | | | |
| --- | --- | --- | --- | --- | --- | --- | --- |
| Gene | Sepal | Spurred Sepal | Petal | Spurred Petal | Reduced Petal | Stamen | Carpel |
| *DeajAP3-1* | - | - | + | - | + | + (s) | +/- |
| *DeajAP3-2* | -/+ | -/+ | -/+ | + | -/+ | + (s) | + |
| *DeajAP3-3* | - | - | + (s) | +(s) | - | +/- | +/- |
| *DeajPI1* | + (s) | + (s) | + (s) | + (s) | + (s) | + (s) | +/- |
| *DeajPI2* | +/- | - | +/- | - | +/- | + | +/- |
| *DeajAGL6-1a* | + (s) | + (s) | + (s) | + (s) | + (s) | +/- | + |
| *DeajAGL6-1b* | + | + | + | + (s) | + | + | +/-/+ |
| *DeajAGL6-2* | + | + | + | + | + | +/- | +/- |

**2.5** *Papaver somniferum* and *Eschscholzia californica*

| *Papaver somniferum* and *Eschscholzia californica* | | | | | |
| --- | --- | --- | --- | --- | --- |
| Gene | Sepal | Petal | Stamen | Carpel | Reference |
| *PapsAP3-1* | +(w) | + | + | +(w) | 13 |
| *PapsAP3-2* | +(w) | + | + | +(w) | 13 |
| *PapsPI-1* | - | + | + | - | 13 |
| *PapsPI-2* | + | + | + (w) | +(w) | 13 |
| *PapsFL-1* | + | + | + | + | 44 |
| *PapsFL-2* | + | + | + | + | 44 |
| *EScaFL1* | + | +(w) | + | +(s) | 44 |
| *EScaFL2* | +(s) | +(s) | +(s) | +(s) | 44 |
| *EScaAG1* | - | - | +(s) | +(s) | 35, 53 |
| *EScaAG2* | - | - | + | +(w) | 35, 53 |
| *DEF1* | - | +(w) | +(w) | - | 35 |
| *DEF2* | - | +(s) | + | - | 35 |
| *DEF3* | - | +(s) | + | - | 35 |
| *SEI* | - | +(s) | + | - | 35 |
| *EScaAGL9* | +(w) | + (s) | + (s) | + | 53 |

**References Cited**

1. Antoń, S., Denisow, B., and Milaniuk, K. (2014). Flowering, pollen production and insect visitation in two *Aconitum* species (Ranunculaceae). *Acta Agrobotanica* 67, 3–12. doi: [10.5586/aa.2014.020](https://doi.org/10.5586/aa.2014.020)
2. Antoń, S., and Kamińska, M. (2015). Comparative floral spur anatomy and nectar secretion in four representatives of Ranunculaceae. *Protoplasma* 252, 1587–1601. doi: [10.1007/s00709-015-0794-5](https://doi.org/10.1007/s00709-015-0794-5)
3. Ballerini, E. S., Kramer, E. M., and Hodges, S. A. (2019). Comparative transcriptomics of early petal development across four diverse species of *Aquilegia* reveal few genes consistently associated with nectar spur development. *BMC Genomics* 20, 668. doi: [10.1186/s12864-019-6002-9](https://doi.org/10.1186/s12864-019-6002-9)
4. Bastida, J. M., Alcántara, J. M., Rey, P. J., Vargas, P., and Herrera, C. M. (2010). Extended phylogeny of *Aquilegia*: the biogeographical and ecological patterns of two simultaneous but contrasting radiations. *Plant Systematics and Evolution* 284, 171–185.
5. Becker, A., Yamada, Y., and Sato, F. (2023). California poppy (*Eschscholzia californica*), the Papaveraceae golden girl model organism for evodevo and specialized metabolism. *Front. Plant Sci.* 14. doi: [10.3389/fpls.2023.1084358](https://doi.org/10.3389/fpls.2023.1084358)
6. Blanché, C. (1990). *Delphinium* L. subgen. *Delphinium*: Origen i tendències evolutives. *Collectanea Botanica* 19, 75–96. doi: [10.3989/collectbot.1990.v19.118](https://doi.org/10.3989/collectbot.1990.v19.118)
7. Bosch, M., Simon, J., Blanché, C., and Molero, J. (1997). Pollination ecology in tribe Delphinieae (Ranunculaceae) in W Mediterranean area: floral visitors and pollinator behavior. *Lagascalia* 19, 545–562.
8. Brunet, J. (2009). Pollinators of the Rocky Mountain columbine: temporal variation, functional groups and associations with floral traits. *Ann Bot* 103, 1567–1578. doi: [10.1093/aob/mcp096](https://doi.org/10.1093/aob/mcp096)
9. Chang, H., Downie, S. R., Peng, H., and Sun, F. (2019). Floral Organogenesis in Three Members of the Tribe Delphinieae (Ranunculaceae). *Plants* 8, 493. doi: [10.3390/plants8110493](https://doi.org/10.3390/plants8110493)
10. Cömert, Y., Tugay, O., and Ulukuş, D. (2023). The morphological, anatomical, palynological, seed micromorphological studies and its taxonomical significance in *Delphinium turcicum* (Ranunculaceae), a local endemic spurless species for Turkey. *BioDiCon*. doi: [10.46309/biodicon.2023.1293145](https://doi.org/10.46309/biodicon.2023.1293145)
11. Cook, S. A. (1962). Genetic System, Variation, and Adaptation in *Eschscholzia Californica*. *Evolution* 16, 278–299. doi: [10.1111/j.1558-5646.1962.tb03220.x](https://doi.org/10.1111/j.1558-5646.1962.tb03220.x)
12. Di Stilio, V. S., Kramer, E. M., and Baum, D. A. (2005). Floral MADS box genes and homeotic gender dimorphism in *Thalictrum dioicum* (Ranunculaceae) – a new model for the study of dioecy. *The Plant Journal* 41, 755–766. doi: [10.1111/j.1365-313X.2005.02336.x](https://doi.org/10.1111/j.1365-313X.2005.02336.x)
13. Drea, S., Hileman, L. C., de Martino, G., and Irish, V. F. (2007). Functional analyses of genetic pathways controlling petal specification in poppy. *Development* 134, 4157–4166. doi: [10.1242/dev.013136](https://doi.org/10.1242/dev.013136)
14. Edwards, M. B., Ballerini, E. S., and Kramer, E. M. (2022). Complex developmental and transcriptional dynamics underlie pollinator-driven evolutionary transitions in nectar spur morphology in *Aquilegia* (columbine). *American Journal of Botany* 109, 1360–1381. doi: [10.1002/ajb2.16046](https://doi.org/10.1002/ajb2.16046)
15. Espinosa, F., Deroin, T., Malécot, V., Wang, W., Pinedo, M., Nadot, S., et al. (2021). Historical note on the taxonomy of the genus *Delphinium* L. (Ranunculaceae) with an amended description of its floral morphology. *Adansonia* 3, 9–18.
16. Espinosa, F., Deroin, T., Xiang, K.-L., Wang, W., Castro, M. P., Byng, J. W., et al. (2017). The Turkish Endemic *Pseudodelphinium Turcicum* (Ranunculaceae): An Unusual Population of *Delphinium* with Peloric Flowers That Has Persisted in the Wild for 20 Years. *International Journal of Plant Sciences* 178, 546–555.
17. Galimba, K. D., and Di Stilio, V. S. (2015). Sub-functionalization to ovule development following duplication of a floral organ identity gene. *Developmental Biology* 405, 158–172. doi: [10.1016/j.ydbio.2015.06.018](https://doi.org/10.1016/j.ydbio.2015.06.018)
18. Galimba, K. D., Tolkin, T. R., Sullivan, A. M., Melzer, R., Theißen, G., and Di Stilio, V. S. (2012). Loss of deeply conserved C-class floral homeotic gene function and C- and E-class protein interaction in a double-flowered ranunculid mutant. *Proceedings of the National Academy of Sciences* 109, E2267–E2275. doi: [10.1073/pnas.1203686109](https://doi.org/10.1073/pnas.1203686109)
19. Geng, F.-D., Xie, J.-H., Xue, C., Sun, L., Li, J.-J., Niu, C.-Y., et al. (2021). Loss of innovative traits underlies multiple origins of *Aquilegia ecalcarata*. *Journal of Systematics and Evolution* 60, 1291–1302. doi: [10.1111/jse.12808](https://doi.org/10.1111/jse.12808)
20. Hidalgo, O., and Gleissberg, S. (2010). Evolution of the reproductive architecture in the bleeding hearts and poppies (Papaveraceae s.l.). *International Journal of Plant Developmental Biology* 4, 76–85.
21. Hodges, S. A. (2015). The Evolution and Ecology of Floral Morphology in *Aquilegia* and the Influence of Horticulture on its Emergence as a Model System. *Acta Horticulturae* 1087, 95–104. doi: [10.17660/actahortic.2015.1087.10](https://doi.org/10.17660/actahortic.2015.1087.10)
22. Hodges, S. A., and Arnold, M. L. (1994). Floral and Ecological Isolation Between *Aquilegia formosa* and *Aquilegia pubescens*. *Proceedings of the National Academy of Sciences of the United States of America* 91, 2493–2496.
23. Hodges, S. A., Fulton, M., Yang, J. Y., and Whittall, J. B. (2004). Verne Grant and Evolutionary Studies of *Aquilegia*. *The New Phytologist* 161, 113–120.
24. Hong, Y., Luo, Y., Gao, Q., Ren, C., Yuan, Q., and Yang, Q.-E. (2017). Phylogeny and reclassification of *Aconitum* subgenus *Lycoctonum* (Ranunculaceae). *PLoS ONE* 12, e0171038. doi: [10.1371/journal.pone.0171038](https://doi.org/10.1371/journal.pone.0171038)
25. Huang, L., Geng, F.-D., Fan, J.-J., Zhai, W., Xue, C., Zhang, X.-H., et al. (2022). Evidence for two types of *Aquilegia ecalcarata* and its implications for adaptation to new environments. *Plant Diversity* 44, 153–162. doi: [10.1016/j.pld.2021.06.006](https://doi.org/10.1016/j.pld.2021.06.006)
26. Jabbour, F., and Renner, S. S. (2012a). A phylogeny of Delphinieae (Ranunculaceae) shows that *Aconitum* is nested within *Delphinium* and that Late Miocene transitions to long life cycles in the Himalayas and Southwest China coincide with bursts in diversification. *Molecular Phylogenetics and Evolution* 62, 928–942. doi: [10.1016/j.ympev.2011.12.005](https://doi.org/10.1016/j.ympev.2011.12.005)
27. Jabbour, F., and Renner, S. S. (2012b). Spurs in a Spur: Perianth Evolution in the Delphinieae (Ranunculaceae). *International Journal of Plant Sciences* 173, 1036–1054. doi: [10.1086/667613](https://doi.org/10.1086/667613)
28. Jabbour, F., Ronse De Craene, L. P., Nadot, S., and Damerval, C. (2009). Establishment of zygomorphy on an ontogenic spiral and evolution of perianth in the tribe Delphinieae (Ranunculaceae). *Annals of Botany* 104, 809–822. doi: [10.1093/aob/mcp162](https://doi.org/10.1093/aob/mcp162)
29. Jabbour, F., Udron, M., Le Guilloux, M., Gonçalves, B., Manicacci, D., Nadot, S., et al. (2015). Flower development schedule and AGAMOUS-like gene expression patterns in two morphs of *Nigella damascena* (Ranunculaceae) differing in floral architecture. *Botanical Journal of the Linnean Society* 178, 608–619. doi: [10.1111/boj.12297](https://doi.org/10.1111/boj.12297)
30. Johns, J. W., Min, Y., Ballerini, E. S., Kramer, E. M., and Hodges, S. A. (2024). Loss of staminodes in *Aquilegia jonesii* reveals a fading stamen–staminode boundary. *EvoDevo* 15, 6. doi: [10.1186/s13227-024-00225-3](https://doi.org/10.1186/s13227-024-00225-3)
31. Kaplan, S. M., and Mulcahy, D. L. (1971). Mode of Pollination and Floral Sexuality in *Thalictrum*. *Evolution* 25, 659–668. doi: [10.2307/2406946](https://doi.org/10.2307/2406946)
32. Kosuge, K., and Tamura, M. (1988). Morphology of the petal in *Aconitum*. *Bot Mag Tokyo* 101, 223–237. doi: [10.1007/BF02488601](https://doi.org/10.1007/BF02488601)
33. Kramer, E. M. (2009). *Aquilegia*: A New Model for Plant Development, Ecology, and Evolution. *Annual Review of Plant Biology* 60, 261–277. doi: [10.1146/annurev.arplant.043008.092051](https://doi.org/10.1146/annurev.arplant.043008.092051)
34. Kramer, E. M., Holappa, L., Gould, B., Jaramillo, M. A., Setnikov, D., and Santiago, P. M. (2007). Elaboration of B Gene Function to Include the Identity of Novel Floral Organs in the Lower Eudicot *Aquilegia*. *The Plant Cell* 19, 750–766. doi: [10.1105/tpc.107.050385](https://doi.org/10.1105/tpc.107.050385)
35. Lange, M., Orashakova, S., Lange, S., Melzer, R., Theißen, G., Smyth, D. R., et al. (2013). The *seirena* B Class Floral Homeotic Mutant of California Poppy (*Eschscholzia californica*) Reveals a Function of the Enigmatic PI Motif in the Formation of Specific Multimeric MADS Domain Protein Complexes. *The Plant Cell* 25, 438–453. doi: [10.1105/tpc.112.105809](https://doi.org/10.1105/tpc.112.105809)
36. LaRue, N. C., Sullivan, A. M., and Di Stilio, V. S. (2013). Functional recapitulation of transitions in sexual systems by homeosis during the evolution of dioecy in *Thalictrum*. *Front. Plant Sci.* 4. doi: [10.3389/fpls.2013.00487](https://doi.org/10.3389/fpls.2013.00487)
37. Liao, H., Fu, X., Zhao, H., Cheng, J., Zhang, R., Yao, X., et al. (2020). The morphology, molecular development and ecological function of pseudonectaries on *Nigella damascena* (Ranunculaceae) petals. *Nat Commun* 11, 1777. doi: [10.1038/s41467-020-15658-2](https://doi.org/10.1038/s41467-020-15658-2)
38. Lu, N.-N., Ma, Y., Hou, M., and Zhao, Z.-G. (2021). The function of floral traits and phenotypic selection in *Aconitum gymnandrum* (Ranunculaceae). *Plant Biology* 23, 931–938. doi: [10.1111/plb.13305](https://doi.org/10.1111/plb.13305)
39. Martínez-Gómez, J., Galimba, K. D., Coté, E. Y., Sullivan, A. M., and Di Stilio, V. S. (2021). Spontaneous homeotic mutants and genetic control of floral organ identity in a ranunculid. *Evolution & Development* 23, 197–214. doi: [10.1111/ede.12357](https://doi.org/10.1111/ede.12357)
40. Meaders, C., Min, Y., Freedberg, K. J., and Kramer, E. (2020). Developmental and molecular characterization of novel staminodes in *Aquilegia*. *Ann Bot* 126, 231–243. doi: [10.1093/aob/mcaa029](https://doi.org/10.1093/aob/mcaa029)
41. Miller, J. a. C., Henning, L., Heazlewood, V. L., Larkin, P. J., Chitty, J., Allen, R., et al. (2005). Pollination biology of oilseed poppy, *Papaver somniferum* L. *Aust. J. Agric. Res.* 56, 483–490. doi: [10.1071/AR04234](https://doi.org/10.1071/AR04234)
42. Morales-Briones, D. F., Arias, T., Di Stilio, V. S., and Tank, D. C. (2019). Chloroplast primers for clade-wide phylogenetic studies of *Thalictrum*. *Applications in Plant Sciences* 7, e11294. doi: [10.1002/aps3.11294](https://doi.org/10.1002/aps3.11294)
43. Ngernsaengsaruay, C., Leksungnoen, N., Chanton, P., Andriyas, T., Thaweekun, P., Rueansri, S., et al. (2023). Morphology, Taxonomy, Anatomy, and Palynology of the Opium Poppy (*Papaver somniferum* L.) Cultivation in Northern Thailand. *Plants* 12, 2105. doi: [10.3390/plants12112105](https://doi.org/10.3390/plants12112105)
44. Pabón-Mora, N., Ambrose, B. A., and Litt, A. (2012). Poppy *APETALA1/FRUITFULL* Orthologs Control Flowering Time, Branching, Perianth Identity, and Fruit Development. *Plant Physiology* 158, 1685–1704. doi: [10.1104/pp.111.192104](https://doi.org/10.1104/pp.111.192104)
45. Pabón‐Mora, N., Sharma, B., Holappa, L. D., Kramer, E. M., and Litt, A. (2013). The *Aquilegia FRUITFULL‐like* genes play key roles in leaf morphogenesis and inflorescence development. *The Plant Journal* 74, 197–212. doi: [10.1111/tpj.12113](https://doi.org/10.1111/tpj.12113)
46. Sharma, B., and Kramer, E. M. (2017). *Aquilegia* B gene homologs promote petaloidy of the sepals and maintenance of the C domain boundary. *EvoDevo* 8, 22. doi: [10.1186/s13227-017-0085-7](https://doi.org/10.1186/s13227-017-0085-7)
47. Sharma, B., Meaders, C., Wolfe, D., Holappa, L., Walcher-Chevillet, C., and Kramer, E. M. (2019). Homologs of LEAFY and UNUSUAL FLORAL ORGANS Promote the Transition from Inflorescence to Floral Meristem Identity in the Cymose *Aquilegia coerulea*. *Front. Plant Sci.* 10. doi: [10.3389/fpls.2019.01218](https://doi.org/10.3389/fpls.2019.01218)
48. Sharma, B., Yant, L., Hodges, S. A., and Kramer, E. M. (2014). Understanding the development and evolution of novel floral form in *Aquilegia*. *Current Opinion in Plant Biology* 17, 22–27. doi: [10.1016/j.pbi.2013.10.006](https://doi.org/10.1016/j.pbi.2013.10.006)
49. Tucker, S. C., and Hodges, S. A. (2005). Floral Ontogeny of *Aquilegia*, *Semiaquilegia*, and *Enemion* (Ranunculaceae). *International Journal of Plant Sciences* 166, 557–574. doi: [10.1086/429848](https://doi.org/10.1086/429848)
50. Vural, M., Duman, H., Aytaç, Z., and Adigüzel, N. (2012). A new genus and three new species from Central Anatolia, Turkey. *Turkish Journal of Botany* 36, 427–433. doi: [10.3906/bot-1105-16](https://doi.org/10.3906/bot-1105-16)
51. Wang, P., Liao, H., Zhang, W., Yu, X., Zhang, R., Shan, H., et al. (2015). Flexibility in the structure of spiral flowers and its underlying mechanisms. *Nature Plants* 2, 15188. doi: [10.1038/nplants.2015.188](https://doi.org/10.1038/nplants.2015.188)
52. Xiang, K.-L., Aytaç, Z., Liu, Y., Espinosa, F., Jabbour, F., Byng, J. W., et al. (2017). Recircumscription of *Delphinium* subg. *Delphinium* (Ranunculaceae) and implications for its biogeography. *TAXON* 66, 554–566. doi: [10.12705/663.3](https://doi.org/10.12705/663.3)
53. Yellina, A. L., Orashakova, S., Lange, S., Erdmann, R., Leebens-Mack, J., and Becker, A. (2010). Floral homeotic C function genes repress specific B function genes in the carpel whorl of the basal eudicot California poppy (*Eschscholzia californica*). *EvoDevo* 1, 13. doi: [10.1186/2041-9139-1-13](https://doi.org/10.1186/2041-9139-1-13)
54. Zahn, L. M., Kong, H., Leebens-Mack, J. H., Kim, S., Soltis, P. S., Landherr, L. L., et al. (2005). The Evolution of the SEPALLATA Subfamily of MADS-Box GenesSequence data from this article have been deposited with the EMBL/GenBank Data Libraries under accession nos. AY850178, AY850179, AY850180, AY850181, AY850182, AY850183, AY850184, AY850185, AY850186. *Genetics* 169, 2209–2223. doi: [10.1534/genetics.104.037770](https://doi.org/10.1534/genetics.104.037770)
55. Zalko, J., Frachon, S., Morel, A., Deroin, T., Espinosa, F., Xiang, K.-L., et al. (2021). Floral Organogenesis and Morphogenesis of *Staphisagria* (Ranunculaceae): Implications for the Evolution of Synorganized Floral Structures in Delphinieae. *International Journal of Plant Sciences* 182, 59–70. doi: [10.1086/711471](https://doi.org/10.1086/711471)
56. Zhang, P., Xie, Y., Xie, W., Li, L., Zhang, H., Duan, X., et al. (2024). Roles of the APETALA3–3 ortholog in the petal identity specification and morphological differentiation in *Delphinium anthriscifolium* flowers. *Horticulture Research* 11, uhae097. doi: [10.1093/hr/uhae097](https://doi.org/10.1093/hr/uhae097)
57. Zhang, W.-G., Liu, Y.-G., Nie, T.-J., Guo, C.-C., Qiu, L.-H., Yang, G.-Y., et al. (2022). Floral ontogeny of *Delphinium anthriscifolium* (Ranunculaceae) and development of intrafloral and symmetrical resupinated organs. *Botanical Journal of the Linnean Society* 198, 86–98. doi: [10.1093/botlinnean/boab041](https://doi.org/10.1093/botlinnean/boab041)
58. Zhao, H., Liao, H., Li, S., Zhang, R., Dai, J., Ma, P., et al. (2023). Delphinieae flowers originated from the rewiring of interactions between duplicated and diversified floral organ identity and symmetry genes. *The Plant Cell* 35, 994–1012. doi: [10.1093/plcell/koac368](https://doi.org/10.1093/plcell/koac368)
59. Zhao, L., Liu, P., Che, X.-F., Wang, W., and Ren, Y. (2011). Floral organogenesis of *Helleborus thibetanus* and *Nigella damascena* (Ranunculaceae) and its systematic significance. *Botanical Journal of the Linnean Society* 166, 431–443. doi: [10.1111/j.1095-8339.2011.01142.x](https://doi.org/10.1111/j.1095-8339.2011.01142.x)
